# Perisaccadic Attentional Updating in Area V4: A Neurocomputational Approach

**DOI:** 10.1111/ejn.70354

**Published:** 2025-12-17

**Authors:** Julia Bergelt, Fred H. Hamker

**Affiliations:** ^1^ Department of Computer Science Chemnitz University of Technology Chemnitz Germany

**Keywords:** attention, eye movement, LIP, spatial perception, V4

## Abstract

Psychophysical studies with human subjects investigated the dynamics of spatial visual attention around the time of saccades. They revealed that attention pointers remap against saccade direction, but at the same time show a lingering at the irrelevant retinotopic position after saccade. Involved brain areas have remained elusive. However, recordings from neurons in visual area V4 of macaques confirm remapping of spatial attention to a position opposite to the saccade direction shortly before saccade onset. Unexpectedly, in comparison with behavioral data from human subjects, a neural correlate of the lingering of spatial attention has not been observed in V4. To better understand the underlying computational mechanisms of attentional updating in V4, we developed a neurocomputational model of perisaccadic visual attention in area V4 by incorporating perisaccadic spatio‐temporal signals from the frontal eye field (FEF) and the lateral intraparietal area (LIP). When we test this model on the same task, our obtained data replicate the observation of predictive remapping of spatial attention pointers in V4. Further, our model provides an intuitive explanation for the lack of lingering attention in the neural recordings: As the monkeys are only rewarded when they detect the target probe at the instructed location, they may aim at minimizing the post‐saccadic spread of attention and thus weaken the attention pointer upon saccade planning. By deactivating different pathways, our model predicts that the attentional enhancement measured in V4 originates from spatial updating of a tonic attention pointer and not from a phasic saccade target attention pointer.

## Graphical abstract

We present a neuro‐computational model to better understand attentional updating during saccades in area V4. The model includes the frontal eye field (FEF), lateral intraparietal area (LIP), and visual area V4. We replicate recent neural cell recordings in macaque's V4 and provide an intuitive explanation of contradictory findings in comparison to behavioral data of human subjects.

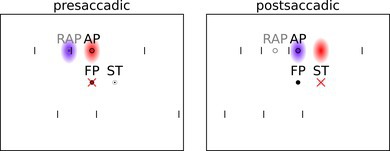

**Abbreviations**
AU: attended to unattended
CD: corollary discharge
FEF: frontal eye field
FP: fixation point
LIP: lateral intraparietal area
PC: proprioceptive
RF: receptive field
ST: saccade target
UA: unattended to attended
UU: unattended to unattended
V1/V4: first/fourth visual area
(R)AP: (remapped) attention position

## 1 Introduction

Humans repeatedly shift their gaze to fixate on and analyze objects of interest, while covert spatial attention allows us to allocate processing resources to other parts of the scene. However, when shifting gaze while keeping attention fixed in retinotopic space, one would attend to a location different than originally planned. Several studies were conducted to investigate the updating of covert spatial attention around eye movements. Studies suggest that attention is shifted against saccade direction already prior to saccade onset (Rolfs et al. 2011), such that after saccade, attention is immediately directed to the original target location. This means that the saccade is anticipated in the sense that attention is remapped predictively in the opposite direction of the saccade, based on the direction and amplitude of the intended movement. The resulting position is called remapped attention position (RAP). Other data show brief post‐saccadic lingering of attention at an irrelevant, retinotopic position before spatial attention is updated to the correct location (Golomb et al. 2008; Golomb 2010). This position arises when the attention pointer is anchored retinotopically and, as a result, shifted according to the saccade vector during the eye movement. Although these update mechanisms may appear contradictory at first glance, Jonikaitis et al. (2013) showed that both phenomena occur simultaneously.

Recently, Marino and Mazer (2018) recorded the activity of neurons in area V4 of two macaque monkeys performing a novel spatiotopic attention task. In initial training trials, a visual cue indicated the location to which spatial attention should be directed in order to perform the task optimally. After those instruction trials, the cue has been omitted, which requires the monkeys to recall the cued location from their memory. In the successive behavioral trials, the monkeys had to execute a guided saccade while ignoring a continuous stream of low‐contrast bars serving as mapping probes and ignoring target shapes with a binary white noise texture appearing at non‐cued locations. If the target appeared at the (previously) cued position, the monkeys had to release a touch bar. While the monkeys performed the task, the activity of 102 neurons in dorsal V4 was recorded to reveal the spatial updating of covert attention around eye movements. Marino and Mazer (2018) analyzed three conditions: In the AU condition, the receptive field (RF) of a neuron moved from the attended (A) to an unattended (U) location, in UA, the RF moved vice versa, and in UU, the RF moved from an unattended to a (different) unattended position. They observed that attentional modulation rises 161 ms before saccade onset in UA trials and decays 85 ms prior to saccade onset in AU trials. In other words, consistent with human studies, they found predictive remapping of spatial attention pointers. Contradictory, their data did not indicate a post‐saccadic lingering of attention in V4. With respect to the potential underlying mechanism, they tested for classical (forward) RF remapping (Duhamel et al. 1992; Marino and Mazer 2016), but did not observe major shifts in RFs. This suggests that their neural signature of updating spatial attention has a different origin.

To explore putative mechanisms of spatial updating in V4, we set up a neurocomputational model of V4 along with external peri‐saccadic spatial signals from the frontal eye field (FEF) and the lateral intraparietal area (LIP). The FEF is well known to display neural signatures of the saccade target (ST) and models predicted that it may affect the attentional state of V4 neurons (Hamker 2005), supported by neural recordings (Moore and Armstrong 2003). LIP has been previously coined as a priority map (Bisley 2011; Bisley et al. 2020) and as such, it may also be involved in the spatial updating of attention. Indeed, we previously demonstrated by means of a neurocomputational model that a corollary discharge (CD) could be used to shift population activity in LIP in an anticipatory fashion to update an attention pointer (Bergelt and Hamker 2019). We ran a simulation of the experiment from Marino and Mazer (2018) and analyzed our model's V4 activity to elaborate which mechanisms are necessary to replicate the experimental data. By deactivating different pathways in the neurocomputational model, we concluded that the attentional enhancement measured in V4 originates from spatial updating (presumably from area LIP) and not from an ST preparation (phasic attention pointer by reentrant processing from FEF to V4). Further, we explore why Marino and Mazer (2018) only observed a neural signature of predictive remapping of attention and no lingering which has been seen in human behavioral data (Golomb et al. 2008; Golomb 2010; Jonikaitis et al. 2013). Our explanations highlight substantial differences in the experimental settings, which may result in different roles for attention in accomplishing the task.

## 2 Materials and Methods

The developed neurocomputational model which is used to simulate the experimental findings of Marino and Mazer (2018) integrates previous, rather separate lines of research: visual attention by reentrant processing (Hamker 2005; Zirnsak et al. 2011; Beuth and Hamker 2015; Beuth 2019) and peri‐saccadic space perception (Ziesche and Hamker 2011, 2014; Ziesche et al. 2017; Bergelt and Hamker 2016, 2019).

**FIGURE 1:**
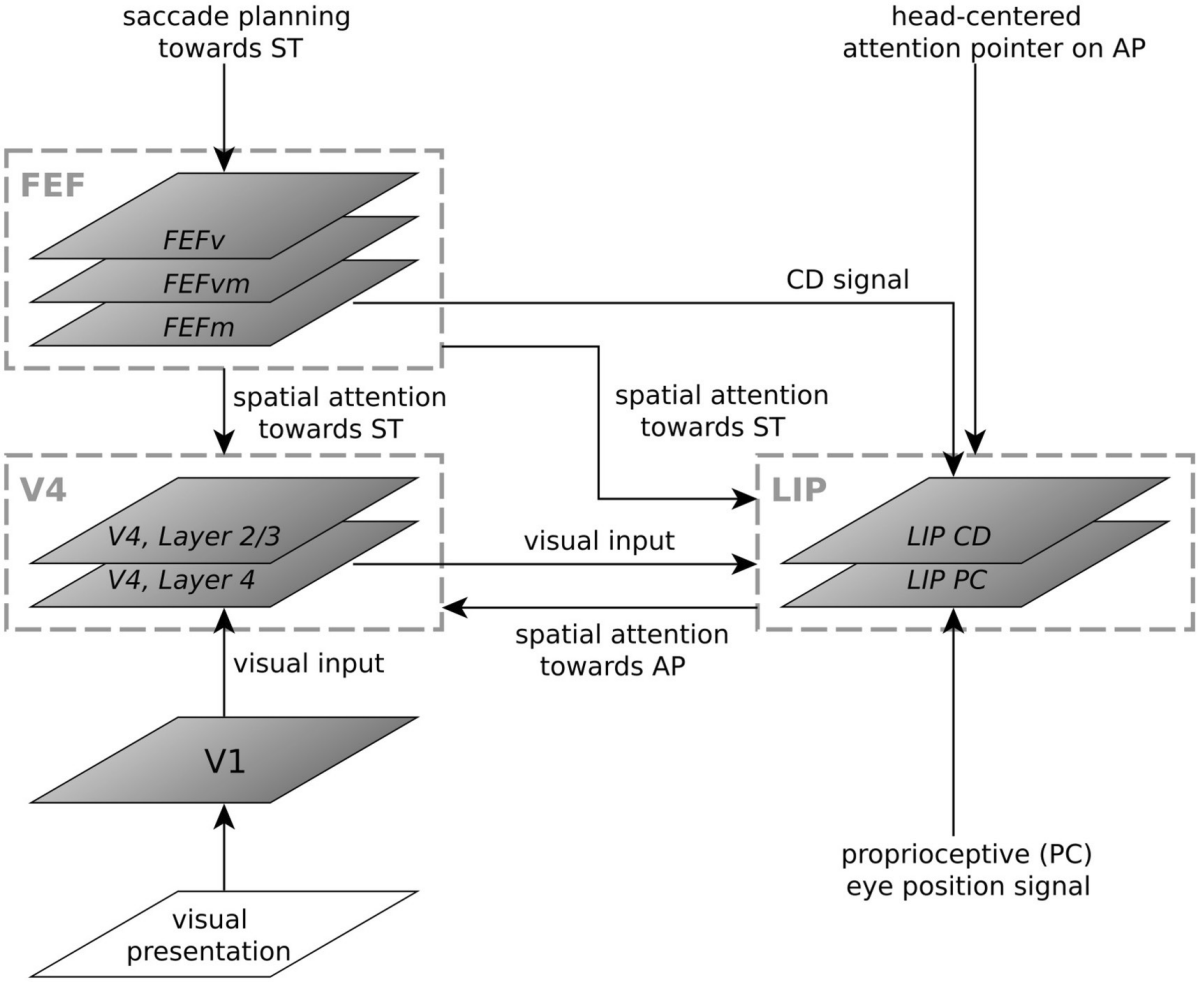
Structure of the neurocomputational model. The model consists of primary and fourth visual cortex (V1 and V4), frontal eye field (FEF), and lateral intraparietal area (LIP). Inputs to the model are the visual presentation of bars (input to V1), time course of a proprioceptive (PC) eye position signal (input to LIP), fixed attention pointer directed to given attention position (AP) in head‐centered coordinates (input to LIP), time course of a corollary discharge (CD) signal (input to FEF) and trajectory of eye position during saccade (from fixation point (FP) to saccade target (ST), used to update the visual presentation due to the eye movement).

The structure of the model is illustrated in Figure 1. It consists of three main parts modeling: (1) primary and intermediate visual cortex (V1 and V4), (2) the FEF, and (3) the LIP. The focus of our model lies on a functional level. Thus, we do not claim to model all biological details but consider main known anatomical pathways.

Obviously, visual area V4 is crucial for the model, as Marino and Mazer (2018) measured the activity of V4 neurons. Following the pathway of the ventral stream (Ungerleider et al. 2008), the visual area V1 is added to pre‐process a visual input given into the model and transferring it to V4. Attentional enhancement in V4 has multiple sources. Especially for spatial attention, the two areas FEF and LIP are of high interest. Moore and Armstrong (2003) showed that FEF has a gain‐modulating influence on V4 neurons that aligns with the eye movement command. Bisley and Goldberg (2003) found a correlation between LIP neuron activity and spatial attention. Moreover, LIP as well as FEF are supposed to be involved in the guidance of attention in visual areas (Bisley 2011) and are considered as “salience maps for spatial attention and maps of potential saccade targets” whose activities serve as “pointers” to target locations (Cavanagh et al. 2010). To comply with these findings, we included these two areas to model spatial attention modulating V4 neurons and elaborate the influence of both on V4 responses.

The model receives five different inputs: (A) visual presentation of bars, (B) time course of a proprioceptive (PC) eye position signal, (C) fixed attention pointer directed to given attention position (AP) in head‐centered coordinates, (D) time course of a CD signal, and (E) trajectory of eye position during saccade. Their usage and role with respect to the model as well as the processing of the inputs in the different model parts will be explained in the following.

### 2.1 V1 and V4

A visual input image sequence containing a continuous stream of flashed bars (Input A) is pre‐processed in visual area V1 and further transferred to area V4. As the visual input is retinotopically organized, it has to be updated with each eye movement by modeling the trajectory of eye position during saccade from fixation point (FP) to ST (Input E) to update the visual presentation accordingly. We here use a rather simple V1 structure as the visual inputs are not complex (simple vertical bars) and there is no need for a differentiation of color or orientation. The visual stimuli are modeled (in accordance with the Marino and Mazer's (2018) experiment) to elicit a response in V1/V4; thus, details of their appearance do not matter.

The visual area V4 is modeled by two layers, Layer 4 receiving the visual input from V1 and Layer 2/3 pooling Layer 4's activity. The structure in V4 has been motivated by modeling experiments of biased competition (Beuth and Hamker 2015; Beuth 2019). As in this task the behavior of both layers is similar, we will report the activity in V4 Layer 4. In addition to the excitatory input from V1, V4 can be modulated by two different signals, by the tonic attention pointer spatially directed to a given AP (here linked to V4 via LIP) and by a phasic spatial attention signal directed towards the ST (here linked to V4 via FEF). This separation of labor regarding top‐down modulatory input is a simplification, but consistent with ideas about functional differences between FEF and LIP (Bisley et al. 2020). Due to the lack of physiological studies in remapping of tonic spatial attention in both brain areas, we hypothesize that LIP is a more likely candidate implementing a tonic attention pointer, as detailed below.

### 2.2 FEF

The FEF plays a crucial role in target selection and saccade generation towards a target (Tehovnik et al. 2000). However, it also feeds back to area V4 and increases the gain of neurons (Moore and Armstrong 2003), thereby implementing a mechanism of spatial attention (Zirnsak et al. 2011). The FEF also shows neural correlates of working memory. For example, V4 projecting FEF neurons show persistent working memory activity during a memory‐guided saccade task (Merrikhi et al. 2017). However, as their activity declines prior to saccade onset, this mechanism may not account for the observation of improved recognition performance at the attended location prior to saccade onset and the lingering of attention after saccade as observed in studies with human subjects (Jonikaitis et al. 2013). Inactivation studies further show that the persistent delay activity in the FEF does not primarily contribute to spatial working memory but rather to eye movement preparation (Jonikaitis et al. 2023). Present findings do not provide convincing evidence that persistent activity in the FEF may implement the functionality of an attention pointer. Further, a strong causal role in maintaining and updating an attention pointer in the FEF may interfere with saccade planning as attention pointers could induce biases for target selection. Thus, in our model we consider the FEF for target selection and phasic attention directed towards the ST and therefore increasing the gain of neurons with RFs around the ST.

The monkeys in Marino and Mazer's (2018) experiment were trained to make a saccade to a specific cued position and did not need to select the ST among different locations or stimuli. Thus, we omit to simulate the saccade preparation in full detail including identification and selection of ST. Instead, we set directly the ST in FEF and do not consider a V4 to FEF projection for ST identification. However, we still model the build‐up and decay of activity at the ST location before and after the saccade (Input D), based on the observation that FEF cells can be characterized as visual, visuomovement, and movement neurons (Lawrence et al. 2005). To mimic this process, we set a realistic rising and decaying activity of FEF visual cells (FEFv) encoding the retinotopic ST position. This spatio‐temporal activity is transferred to FEF visuomovement cells (FEFvm) and from them to FEF movement cells (FEFm). The activity of FEFm can be interpreted as an eye‐centered CD signal needed for further processing in LIP.

FEFvm projects to V4 modulating its activity, as motivated on the basis of covert attention experiments (Thompson et al. 2005) such that saccade preparation in FEF increases the gain of those neurons in V4 with their RF around the ST. Even though the FEF also shows perisaccadic RF shifts (Zirnsak et al. 2014; Wang et al. 2024), for simplicity, we only model the FEF with respect to its involvement in directed spatial attention towards the ST (Moore 2006).

### 2.3 LIP

Compared to the FEF, the LIP is more involved in providing a task‐specific map of the visual world (Mirpour and Bisley 2021; Sapountzis et al. 2018). Even though LIP receives input from multiple brain areas, we consider the task‐specific activity of V4 projecting to LIP in our model. It is gain modulated by two different eye‐position related signals to produce a combined representation of stimuli and eye related signals using radial basis‐function maps (RBF; for modeling RBFs please refer to Pouget et al. 2002). The first eye related signal is a PC eye position signal (Input B), which encodes the eye position in a head‐centered reference frame and updates after saccade end, inspired by observations in the primary somatosensory cortex (Wang et al. 2007) or in the central thalamus (Tanaka 2007). Evidence suggests that LIP gain fields update to the new eye position late after saccade (Xu et al. 2011), although Morris et al. (2016) have been able to decode a correct continuous eye position signal from LIP population activity. We explained this potential discrepancy on the basis of neurocomputational simulations, according to which late updating LIP gain fields reflect the influence of the modulatory PC signal and LIP population activity includes both modulatory signals, PC and CD (Stocks and Hamker 2025). In our model, the PC signal starts to update 60 ms after the saccade finished. As the second eye related signal, we use a CD signal that may arise from the superior colliculus (SC) further projected via the mediodorsal thalamus into FEF (Sommer and Wurtz 2004). We use the (retinotopic) activity of FEFm, convert it into a head‐centered CD signal (using coordinate transformation with RBFs), and feed it into LIP. For the purpose of easier modeling and evaluation, LIP is (artificially) split into two parts according to the modulating eye related signal (LIP PC and LIP CD, respectively).

LIP further receives an endogenous attention pointer to mimic the indication of the spatiotopic location (Input C) as done in the initial introduction trials in the study of Marino and Mazer (2018). As further discussed in the results section, we modeled two different types of the endogenous attention signal: One type is a tonic attention signal, which is active for the entire simulation of a trial, while the other type is a phasic signal, which is initially active in each trial but deactivated before and around saccade onset. The endogenous attention pointer interacts with the two eye‐related signals (PC and CD signal). This results in two attention pointers in LIP, as demonstrated by Bergelt and Hamker (2019). The attention pointer generated through the PC signal in combination with visual stimulation or top‐down attention results in an attentional enhancement at the attended position. However, due to the late‐updating eye position signal, the pointer moves with the eye movement to the irrelevant, retinotopic position known as the (attentional) lingering effect. The peri‐saccadically active CD signal induces a pre‐saccadic attentional pointer at the remapped attended position which shifts with a saccade to the originally attended position, i.e., remapping of attention.

Finally, LIP neurons encoding the ST also receive input from FEFvm on the lines of the FEFvm‐to‐V4 projection to enhance the activity of neurons related (spatially) to the ST.

Consistent with the idea of LIP as a priority map (Bisley 2011), the LIP activity projects to V4, where it increases the gain of those neurons in response to the presentation of a bar.

The model, including all differential equations, is explained in greater detail in the S2. Details of the neuro‐dynamics are illustrated in a movie (see Video S2).

### 2.4 Experimental Setup

We closely replicated the experimental setup of Marino and Mazer (2018) to be presented to our neurocomputational model. In a visual field of 40° × 30° with point of origin at the center of the visual array, the FP is set at the center (0°*,*0°). The ST is fixed to 5° to the right of FP. The eye movement is initiated 350 ms after the start of the simulation and the saccade trajectory is determined using the model of Van Wetter and Van Opstal (2008) which leads to a horizontal saccade lasting 67 ms. In contrast to Marino and Mazer's (2018) setup, we can use fixed positions for FP and ST as our model has no memory of previous eye movements like monkeys have. The position to be attended (AP) is either above or below FP or ST resulting in four possible positions. The visual input stream consists of randomly flashed bars above and below the horizontal center line of the visual array with horizontal positions randomly distributed over the whole width of the visual array (with some constraints to avoid clustering of stimuli in one area). The bars are flashed between 300 ms before and 300 ms after saccade onset for a random duration between 5 and 20 ms. The total simulation time was 700 ms per trial. The target presentation as well as a decision process modeling the monkey's answer to the target is not simulated as only the recordings in V4 triggered by the probe stimuli are of interest in this study. In the experimental study this task only served as a measure of attention allocation.

**FIGURE 2:**
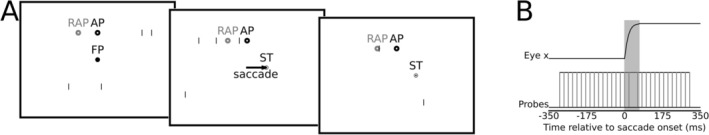
Example of simulated trials. The center of the visual field (FP) is fixated for 350 ms (left panel in A, upper line in B). A saccade is executed 5° to the right which lasts 67 ms (middle panel in A, gray bar in B). Afterwards, the eyes remain at the saccade target (ST; right panel in A, upper line in B). Between 300 ms before and 300 ms after saccade onset, bars are randomly flashed above and below the horizontal center line of the visual array (lower line in B). The attention position (AP) can be located at 4 different positions (here: above FP) and is set at the beginning of each trial. Additionally, the remapped attention position (RAP) is shown in gray in this figure, even though this position has not been explicitly modeled in any of the simulations.

In total, we simulated 4000 different trials to compensate for the randomness in AP and visual input stream. Counting the total number of all combinations of bar position and onset that were created within those trials, separated for the four different APs and split between bars presented above and below the horizontal center line, reveals that all possible combinations are covered at least four times. One example trial including spatial and temporal layout is illustrated in Figure 2. For a detailed visualization of the inputs over time, please refer to the movie (see Video S2).

## 3 Results

**FIGURE 3:**
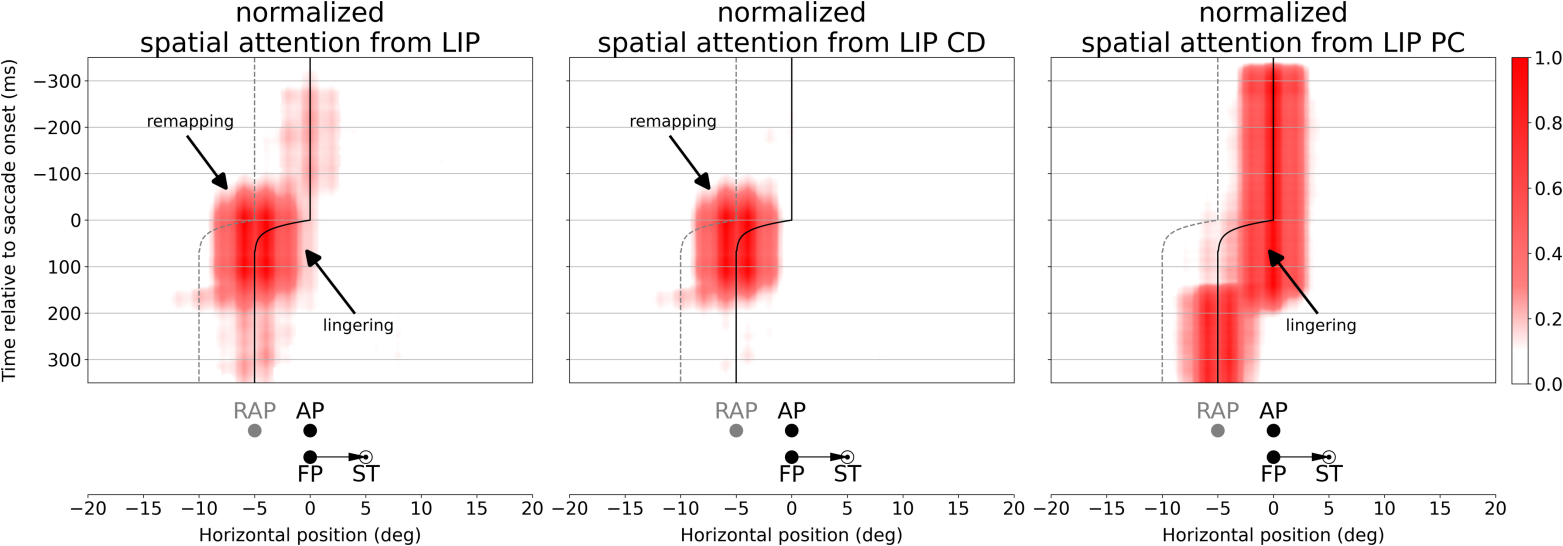
Gain‐modulating activity projected to V4 from LIP CD (middle) and LIP PC (right) as well as their sum (left) over time. As the saccade is only horizontal, the vertical component can be neglected and is therefore fixed in this figure. The time (in milliseconds) is aligned to saccade onset. The vertical solid line represents the ideal attention position (AP) prior, during and after saccade while the dashed line indicates the remapped attention position (RAP). Note, that the projected activity is retinotopically organized, thus the encoded position of a neuron shifts according to the eye movement, i.e., after the saccade originally RAP encoding neurons now encode AP. The weighted neural activity is averaged over all runs with the same given attention position (above fixation point, see, e.g., Figure 2A). We plotted activity of V4 Layer 4, as Layer 2/3 contains the same information but in reduced resolution, due to spatial pooling.

We applied our model to the trans‐saccadic, spatiotopic attention task originally developed by Marino and Mazer (2018) for recordings in monkey area V4. The monkeys were trained to attend to a particular location in space, the AP. The particular novelty of this task is its aim to test trans‐saccadic changes of AP related activity in area V4. Thus, we set a tonic attention pointer directed to AP in the LIP part of the model. After simulation (see Video S1) and data collection, we first analyzed the signal reaching V4 from LIP. Figure 3 shows the gain‐modulating input activity to V4 neurons along the horizontal axis of visual space^1^ over time with respect to its originating LIP part (LIP PC and LIP CD). The solid and dashed lines mark ideal AP and RAP in retinotopic space and their changes due to the eye movement. Like V4 itself, the projected activity to V4 is also organized retinotopically. Thus, the neuron encoding AP pre‐saccadically is different from the neuron encoding AP post‐saccadically (same for RAP, illustrated by the lines in Figure 3). When we look at the contribution from LIP cells projected to V4 separately, we see a pre‐saccadically remapped attention pointer from LIP CD and a late update of the attention pointer from LIP PC. Before saccade onset, AP encoding neurons receive activity from LIP PC (Figure 3, right panel). The CD signal becomes active prior to the saccade and is directed to the ST, i.e., the future eye position. The interaction of the CD signal with top‐down attention leads to an attention pointer in LIP CD directed to neurons at RAP before saccade onset (Figure 3, middle panel). After saccade, AP is encoded by the neurons which had encoded the RAP before saccade onset (see solid line in Figure 3). Thus, the AP encoding neurons immediately receive activity from LIP CD. The activity from LIP PC is projected to neurons encoding an irrelevant, lingering AP following the eye movement as the PC signal is not yet updated and still encodes the pre‐saccadic eye position. With the update to the post‐saccadic eye position, the activity in LIP PC updates as well and projects again to the AP encoding neurons. The update of the PC signal appears to be jump‐like after saccade end (Wang et al. 2007), that is disappearing at the pre‐saccadic position and appearing at the post‐saccadic eye position. As a result, the LIP PC attention pointer also “jumps” from the (irrelevant) lingering position to the originally attended location and replaces the fading LIP CD attention pointer without activating intermediate locations between these two positions. See also Bergelt and Hamker (2019) for a detailed description of the proposed origin of remapping and lingering of attention in LIP.

**FIGURE 4:**
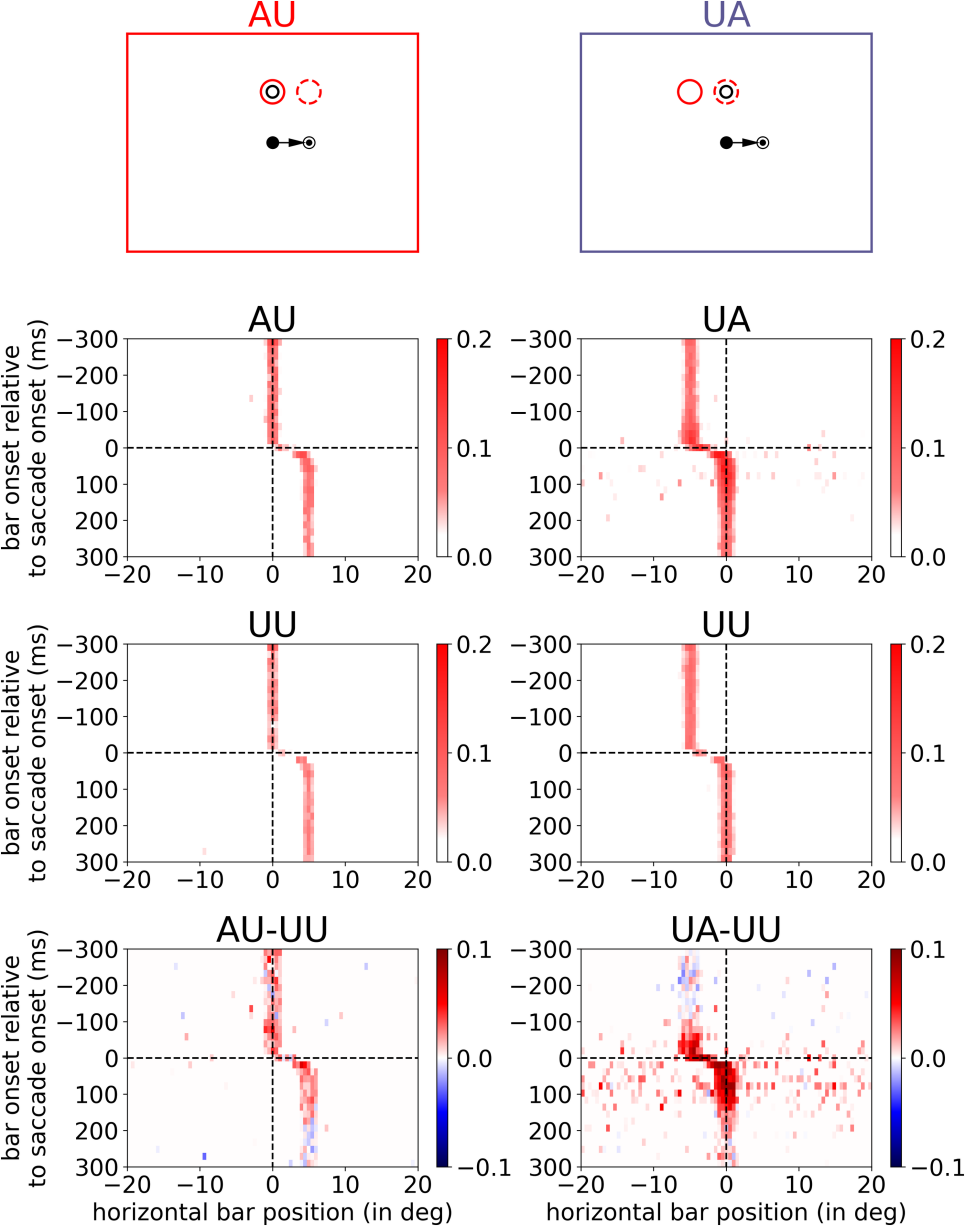
Reverse correlation of V4 responses for combinations of presented bar stimuli with a specific attention position (above fixation point) for one example neuron in the AU condition (left) and one in the UA condition (right). The topmost row visualizes the two tasks (AU and UA) with the position of the neuron's receptive field before (solid red circle) and after (dashed red circle) saccade as well as the cued attention position (black circle). The second row visualizes the neuron's responses in AU and UA conditions. The third row shows the responses in the UU conditions of these neurons. The difference in activity between attention and control task (AU/UA compared to UU) is plotted in the bottom row. For better visualization we applied noise reduction to the responses by subtracting a fixed threshold (equal to the half of maximum response in UU) and bounding the responses to zero.

Next, we calculated the attentional enhancement of V4 neurons (again using the neurons in Layer 4) by reverse correlation of the neural responses to all presented bar stimuli. Figure 4 shows the results for one specific AP (namely, above the FP) and neuronal responses corresponding to the two attention conditions (AU in left column, UA in right column). The position of the neuron's RF is illustrated by a red circle, topmost row (solid circle for pre‐saccadic position, dashed circle for post‐saccadic position). The response of each neuron is averaged over a time window of 20 ms starting with bar onset similar to the sliding window used by Marino and Mazer (2018). The responses of the same neuron chosen for AU and UA tasks, respectively, are shown in Figure 4, third row. There, a (control) UU task is performed, where the cued attention lies at the vertically opposite position. The difference between an attention task (AU or UA) and the corresponding control task (UU) is plotted in the lower row of the figure, which visualizes the attentional enhancement for this specific neuron. The neuron in the AU task shows an enhanced response from the beginning of the simulation, due to the attention condition. However, this enhanced response extends beyond saccade, although it now encodes an irrelevant position. In the UA task, attentional enhancement starts prior to saccade onset. This enhancement persists after saccade, where the neuron encodes now the given AP. Thus, in the UA task the neural response anticipates the state of attention with respect to the eye movement.

**FIGURE 5:**
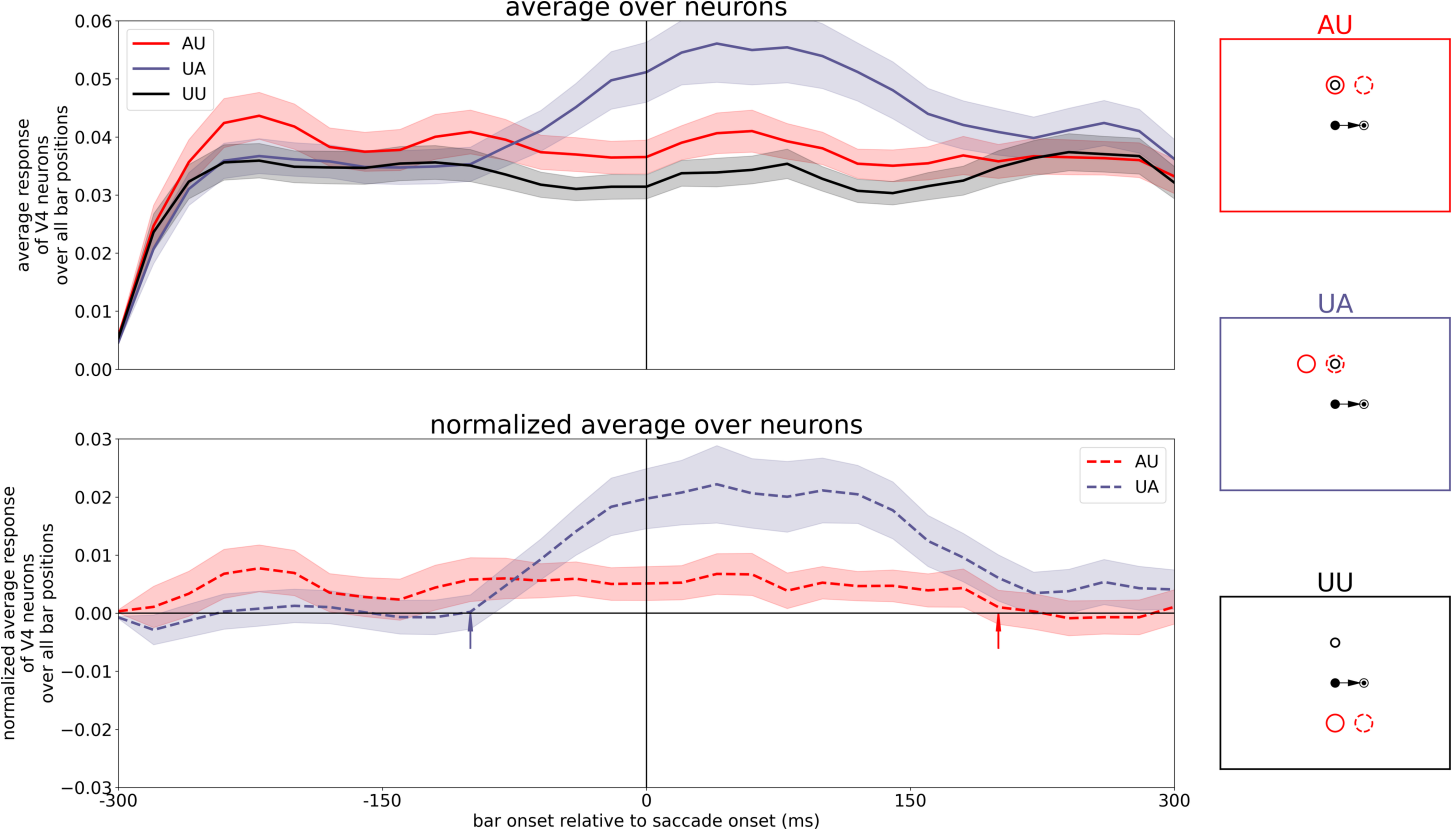
Averaged activity of V4 neurons for the three different tasks (AU, UA, and UU as illustrated by the red, purple, and black boxes on the right) at the top while the plot at the bottom shows the response of neurons in AU and UA task normalized with UU task. The arrows mark offset of attention enhancement in the AU task (red) and onset of attentional enhancement in the UA task (purple). The colored band around the lines illustrate the variance within the different trials.

By evaluating the neuronal activity in a similar manner as Marino and Mazer (2018) (compare their Figure 4B), we reveal the averaged activity of all model V4 neurons for the three different tasks. We calculated the mean activity of the V4, Layer 4 neurons matching the task conditions (AU, UA, or UU) over 20 ms time windows and averaged it over all trials (shown in Figure 5, upper plot). To extract the attentional enhancement in the attention tasks, we subtracted the averaged activity of the UU task from the AU and UA task, respectively (normalized activity, Figure 5, lower plot). In addition, we calculated the time point where the normalized activities in both tasks (AU and UA) had changed. In the AU task, this means a decrease of activity towards zero, and in the UA task, a rise of activity from zero to a positive value. Starting from the beginning of the simulation, we test if the normalized V4 activity in each time window is significantly greater than 0 (*t*‐test, *p >* 0.05). When the activity of three consecutive time windows is not significantly greater than 0 anymore in the AU task, we define this time point as “offset in AU.” Similarly, when the mean activity of three consecutive time windows is significantly greater than 0 in the UA task, we have the “onset in UA.” Like in the experimental results, the attentional modulation in the UA task starts before saccade onset (approximately 100 ms before saccade onset, marked with purple arrow) and rises continuously. But in contrast to the experimental findings, the attentional modulation in AU persists late after saccade onset (up to 200 ms after saccade onset, marked with red arrow). That means our neurocomputational model using tonic top‐down attention as an input generates both (predictive) remapping and lingering of attention while the experiment of Marino and Mazer (2018) shows only remapping of attention. Thus, the model with a permanent top‐down attention pointer is not consistent with the observation of Marino and Mazer (2018), as they did not observe a neural correlate in V4 for attentional lingering. However, the model is consistent with at least four psychophysical studies that observed a correlate of attentional lingering (Golomb et al. 2008; Golomb 2010; Mathôt and Theeuwes 2010; Jonikaitis et al. 2013).

### 3.1 Robustness of the Model

**FIGURE 6:**
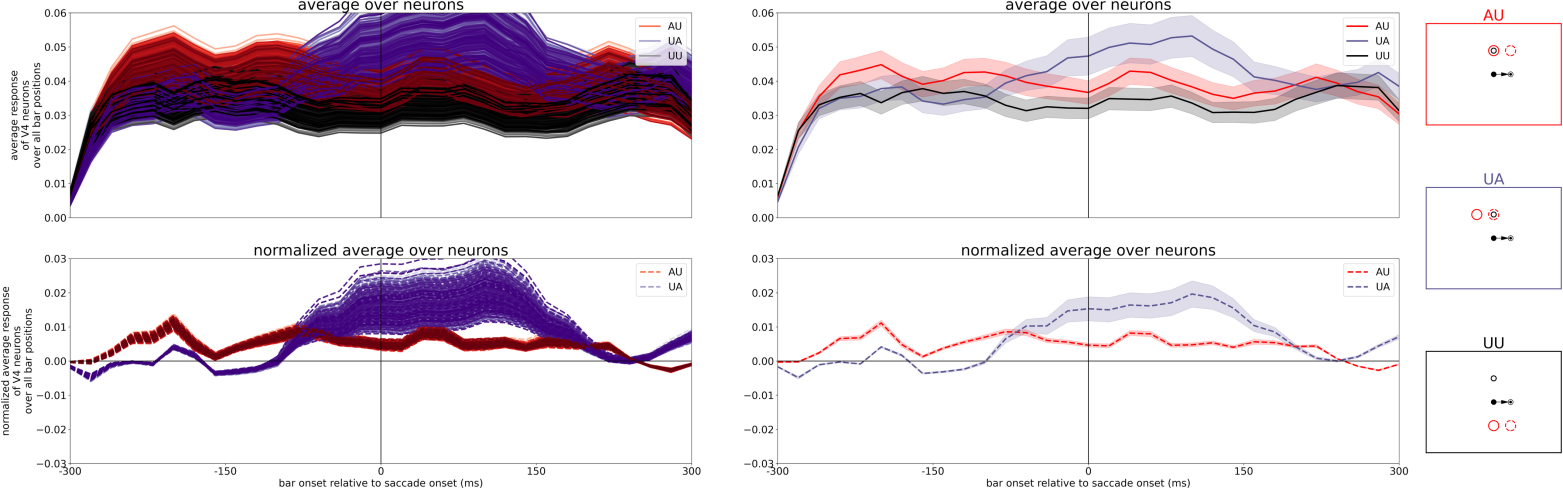
Left: Individually averaged activity of V4 neurons for the three different tasks (AU, UA, and UU as illustrated on the right) at the top while the plot at the bottom shows the response of neurons in AU and UA task normalized with UU task for all parameter sets. Right: Mean and standard deviation of averaged activity of V4 neurons over all parameter sets.

To test the robustness of the model, we changed 60 parameters randomly by ±5% of the original value, simulated the experiment and evaluated the activity of V4 neurons. The chosen parameters relate to the ordinary differential equations of the neurons in the neuro‐computational model as well as the connections between the different maps. A list of the changed parameters can be found in the Supporting Information. In total, we simulated the experiment nearly 700 times, each simulation with a different parameter set. To speed up the total simulation time, we used only trials with an AP above the FP resulting in 998 trials per experiment. In a pre‐test, we verified that this procedure provides reliable results for the evaluation of the activity of the V4 neurons.^2^ Figure 6 shows the individual averaged activities of V4 neurons for the different tasks for all parameter sets (left) as well as the mean and standard deviation over all parameter sets (right). The parameter variation results mainly in a quantitative deviation of the normalized V4 activity in the UA task. However, the qualitative course of the activities in all tasks is sufficiently stable. Thus, even when the parameters defining the main parts of the model (ODEs and connection pattern) are (slightly) varied, the results stay the same, i.e., the model is robust with respect to its main parameters.

### 3.2 Differences of Attentional Modulation

Before we discuss the potential differences of the Marino and Mazer (2018) study to previous studies in humans (Golomb et al. 2008; Golomb 2010; Mathôt and Theeuwes 2010; Jonikaitis et al. 2013), we test our model with respect to its prediction about the cause of the spatial attentional enhancement in V4. Therefore, we simulated the same experiment but with disabling parts of the model. In our model, there are two sources of spatial attentional enhancement, namely, ST directed phasic attention (here simplified via FEF only) and backward remapping of attention pointers (here simplified via LIP only).

**FIGURE 7:**
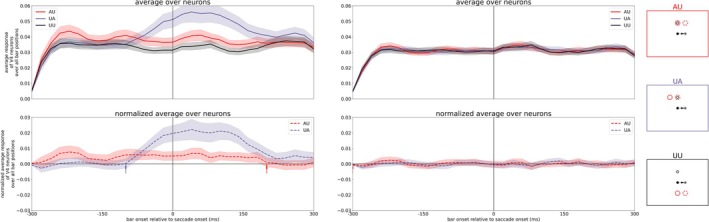
Averaged activity of V4 neurons for the three different tasks (AU, UA, and UU as illustrated by the red, purple, and black boxes on the right) at the top while the plot at the bottom shows the response of neurons in AU and UA task normalized with UU task with deactivated pathways: Deactivating the connection from FEF to V4, i.e., removal of saccade target directed attention (left) and deactivating the connection from LIP to V4, i.e., removal of backward remapping of attention pointers (right).

First, we removed ST directed attention by cutting the connection from FEF to V4 (Figure 7, left). Second, we removed backward remapping of attention pointers by cutting the connection from LIP to V4 (Figure 7, right). Our results show that only the removal of backward remapping of attention pointers affects the update of attention (compare Figure 7 with Figure 5). The underlying reason is that the FEF provides an attentional signal to the planned location of the ST unrelated to the cued AP. The removal of backward remapping of attention pointers leads to a decline in attentional enhancement in V4 for any of the attentional tasks (AU and UA). Thus, the spatial attention effects in our model V4 originate from remapping of attention pointers which is in our model ascribed to LIP. Of course, both LIP and FEF show ST related attention and remapping (Wang et al. 2024) and they may operate in a network rather than having fully distinct roles. However, the FEF together with the SC appears to play a more critical role in providing a CD signal to LIP, enabling remapping of spatial attention.

In simpler terms: As the probes are located only above and below the ST, but never at the ST itself, attentional enhancement from FEF (which is directed towards ST) cannot affect the neuronal responses of those neurons. On the other hand, LIP induced attention is located on the same (horizontal) line as the presented stimuli as the original cued attention is located there. Thus, it can influence these neurons.

### 3.3 Missing Lingering Effect in V4

After determining the cause of spatial attentional enhancement in V4, we now investigate the missing lingering effect in Marino and Mazer's (2018) results, given that several other studies like Golomb et al. (2008), Golomb (2010), Mathôt and Theeuwes (2010), or Jonikaitis et al. (2013) observed lingering in their behavioral data with humans.

**FIGURE 8:**
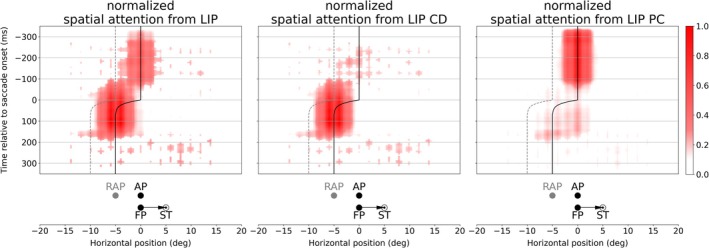
Gain‐modulating activity from LIP CD (middle) and LIP PC (right) projected to V4 as well as their sum (left) over time with fading top‐down attention. Symbols and definitions identical to Figure 3.

**FIGURE 9:**
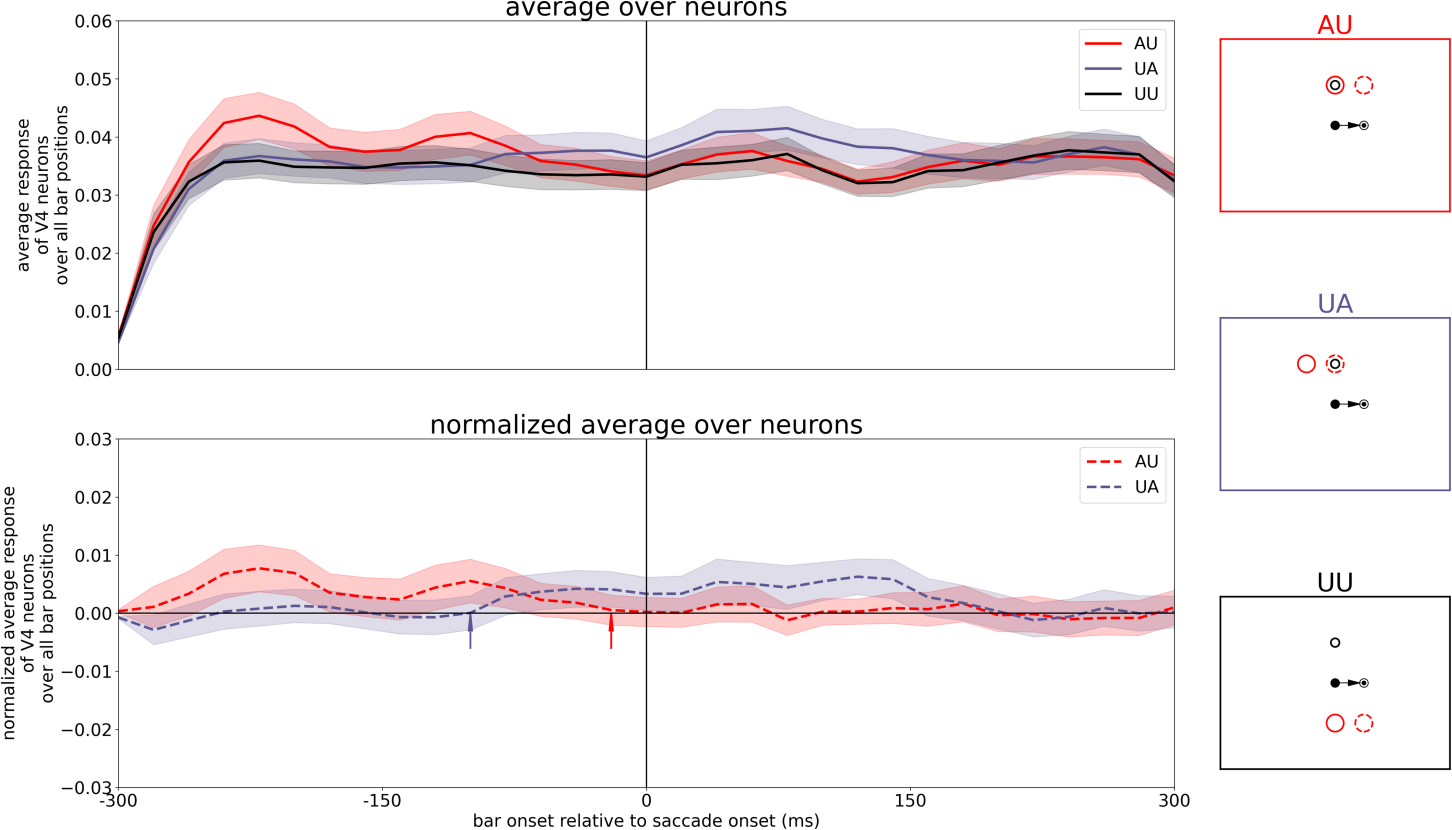
Averaged activity of V4 neurons for the three different tasks (AU, UA, and UU as illustrated on the right) at the top while the plot at the bottom shows the response of neurons in AU and UA task normalized with UU task with fading top‐down attention.

We were wondering about the putative differences between these studies, despite the different species. It may be that the different experimental setups resulted in a slightly different behavior. Jonikaitis et al. (2013) conducted two versions of attentional cuing: one with a permanent attentional cue and one with a salient onset of an irrelevant color cue in each trial. Also, the other studies used explicit cues to guide attention in each trial. In contrast, the monkeys in Marino and Mazer (2018)’s experiment had to perform initial instruction trials before the actual task to learn establishing an endogenous attention signal to a given position. During the task itself, there was no attentional cue any more. Further, the stimulus load is different. The mapping of RFs in Marino and Mazer's (2018) experiment required a large number of probe stimuli which can be attentionally distracting due to the random flashes at various positions over the whole visual scene. Visuo‐spatial attention without a bottom‐up cue is a cognitively demanding task and can be easily disrupted by flashing stimuli (Schreij et al. 2008; Grubb et al. 2015; Demeter and Woldorff 2016). A further major difference, and perhaps the primary reason for the differing observations, lies in the role of attention to accomplish the task. Jonikaitis et al. (2013) instructed their subjects to report a target stimulus that appeared at 6 different locations with equal probability. Under these conditions, attention distributed to different locations may not be harmful and could even result in an advantage. Monkeys in Marino and Mazer's (2018) experiment received reward only when they released a touch bar upon the appearance of the target at the instructed target location. If the target appears at any other location, monkeys were required to continue holding the touch bar. Moreover, following each error a timeout period was triggered, in which monkeys were again instructed about the target location by a visual cue. Under these conditions, a good strategy may be to initially start with an attention pointer directed to the target location but then switch it off to minimize the spread of attention in the post‐saccadic lingering period. Thus, we re‐ran the experiment with an endogenous attention pointer set at the beginning of each trial, but let it fade after variable times (−150, −100, −50, and 50 ms relative to saccade onset). Figures 8 and 9 show the most consistent results with a top‐down attention pointer fading at −100 ms. One can see a reduced activity for the AP encoding neuron in LIP PC starting around 80 ms before saccade onset (compare Figures 3 and 8). The reduced activity in LIP results in a faster decay of attentional modulation in the AU task which now already vanishes prior to saccade onset (around 20 ms before saccade onset, compare Figures 5 and 9). In contrast to the vanishing lingering effect, remapping of attention is still present.

**FIGURE 10:**
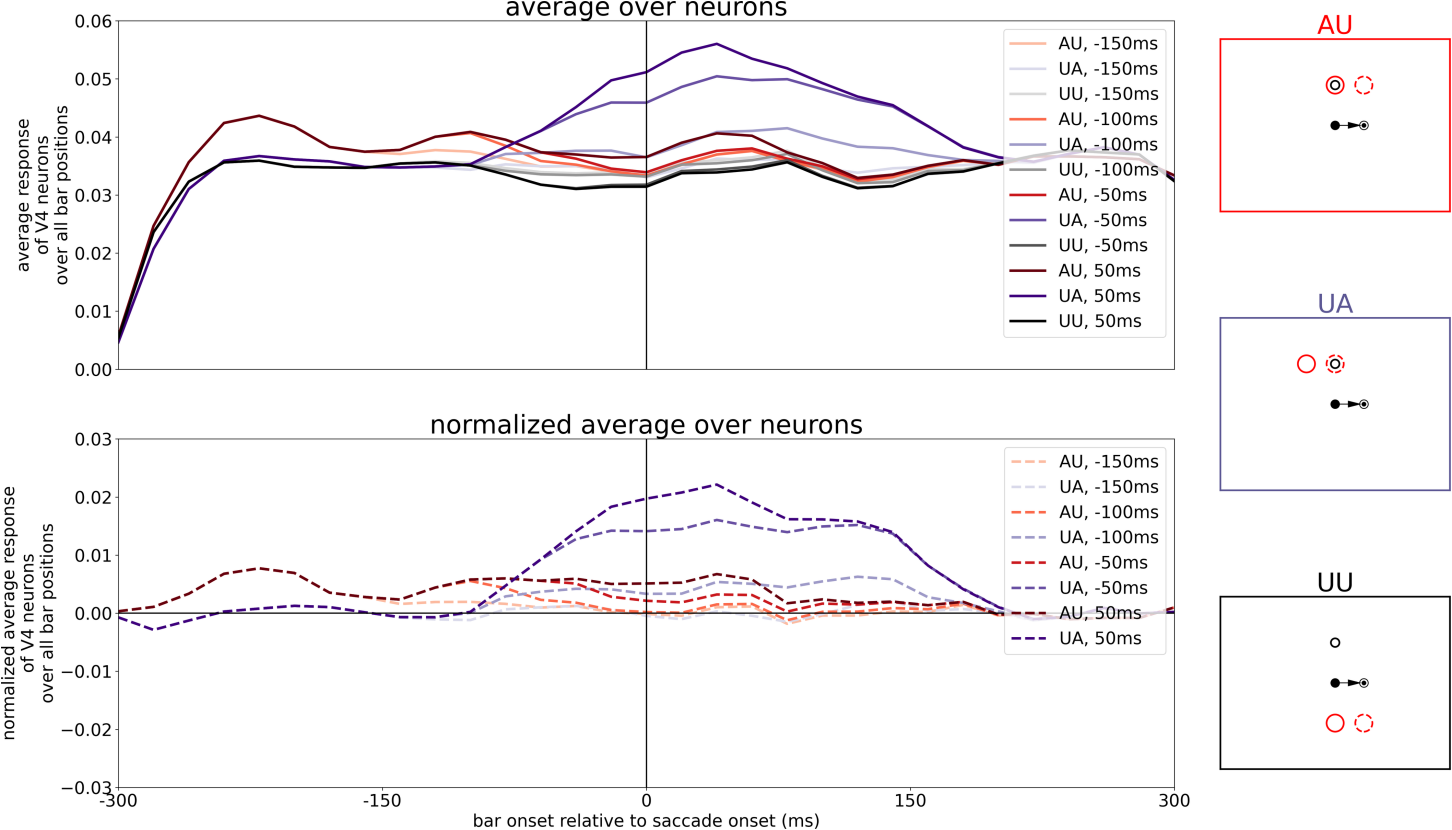
Averaged activity of V4 neurons for the three different tasks (AU, UA, and UU as illustrated by the red, purple, and black boxes on the right) at the top while the plot at the bottom shows the response of neurons in AU and UA task normalized with UU task for different time points of turning off top‐down attention (−150, −100, −50, and 50 ms relative to saccade onset).

The resulting V4 activities of runs with the other offset times are shown in Figure 10. Additionally, Table 1 summarizes the calculated time points of state change in the AU and UA task for the different offset times of the top‐down attention input. If the top‐down attention is turned off too early (e.g., already 150 ms before saccade onset), the attention pointer does not have enough time to build up together with the CD signal a remapped attention pointer as the saccade is not yet in planning and thus the CD signal has not yet risen. If the top‐down attention input is longer active (at least until 100 ms before saccade onset), the remapping effect can be established. However, if the input is turned off too late, the lingering effect can be established as well as the attention pointer is then manifested in the system, resulting in similar activity patterns as for a tonic attention input.

**TABLE 1:** Time points of state change in AU and UA task relative so saccade onset for different time points (relative to saccade onset) of turning off the top‐down attention input.

Turning off time of top‐down attention input	−150 ms	−100 ms	−50 ms	50 ms
Offset in AU	−140 ms	−20 ms	200 ms	200 ms
Onset in UA	—	−100 ms	−100 ms	−100 ms

Concluding, a fading attention pointer to avoid the post‐saccadic spread of attention may be a possible explanation for the discrepancy between observations by Marino and Mazer (2018) and previous studies with humans (Golomb et al. 2008; Golomb 2010; Mathôt and Theeuwes 2010; Jonikaitis et al. 2013).

## 4 Discussion

Many experiments have been conducted to explore the relation between eye movements and visual attention. Early work found an increase of attention at the ST location prior to saccade onset (Deubel and Schneider 1996; Rolfs et al. 2011). Such presaccadic attention has been explained by computational models that implement a re‐entrant connection between build‐up neurons in saccade preparation areas such as the FEF and visual areas (Hamker 2005; Hamker et al. 2008). Such anatomical design is functionally meaningful, as in most cases the content at the ST is likely relevant for the task at hand and by this mechanism, pre‐processed prior to fixation (Hamker et al. 2008). However, at the same time, the visual system can also establish covert attention pointers. Cavanagh et al. (2010) reasoned that such a pointer may update (remap) prior to saccade to establish attention at the veridical location already when the eyes land. Results from Rolfs et al. (2011) supported this hypothesis, while other studies found attentional lingering at the task‐irrelevant location after saccade (Golomb et al. 2008; Golomb 2010; Golomb et al. 2011; Mathôt and Theeuwes 2010). This may appear conflicting, but data from Jonikaitis et al. (2013) suggest that attentional remapping, that is a shift of attention against saccade direction, and attentional lingering, a shift of attention with the saccade, occur at the same time. Even if attention appears temporally distributed across different locations, target performance does not appear to be impaired by distractors present at lingering or remapped locations (Yao et al. 2016). Marino and Mazer (2018) designed a novel paradigm for mapping the state of attention in area V4 in macaque monkey before, during, and after saccade to study the physiological correlate of attention during saccade.

In order to better understand the underlying mechanisms of attention in V4, we designed a model according to which V4 receives bottom‐up input from V1 and modulatory input from LIP and FEF. Our modeling study supports the idea that remapping in LIP can be responsible for the V4 attention effects, even though both LIP and FEF display correlates of remapping (Bisley et al. 2020; Wang et al. 2024), such that we do not explicitly rule out a role of the FEF. However, previous physiological studies of remapping did not investigate the remapping of attention pointers, so that the exact brain's location of this mechanism is unknown. As described in Bergelt and Hamker (2019), LIP may generate two separate attention pointers, one driven by a late updating (PC) eye position signal and one by a phasic CD. In our present study, we observe that around saccades, both pointers exist in parallel, enhancing V4 activity at two different spatial positions: before the eyes move, the spatiotopic AP as well as the RAP receive modulating input from LIP. As the modulating attention pointers from LIP are retinotopic, the attended locations are shifted along with the saccade to an irrelevant, retinotopic AP but at the same time to the originally attended location, respectively. After the eyes had landed, the attention pointer lingering at the irrelevant position jumps back to the originally attended location following the updating of the eye position signal. During the update, no intermediate positions are attended, as observed in Golomb et al. (2011). The idea of two attention pointers existing in parallel is consistent with Golomb's dual‐spotlight theory of attentional updating (Golomb 2019). It suggests a fast, predictive stage where spatial attention is actively and presumably task‐dependently updated to the new location, and a slow, post‐saccadic stage where spatial attention is canceled at the previous retinotopic location through an “automatic, passive decay of neural activity” (Golomb 2019). Importantly, these two stages do not have to be synchronized, so that there might be a period of time where both stages are active in parallel.

Our model generates predictive remapping as well as lingering of attention at the task irrelevant location after saccade. Although this is consistent with previous findings in multiple psychophysical studies with humans (Rolfs et al. 2011; Golomb et al. 2008; Golomb 2010; Mathôt and Theeuwes 2010; Jonikaitis et al. 2013), it is different from the observations of Marino and Mazer (2018) who did not find a neural correlate of lingering in V4. We tested our model with a fading attention pointer and could replicate the data of Marino and Mazer (2018) under this condition. Several reasons suggest a fading attention pointer being a likely cause. First, different from the studies with humans where attention has been guided in each trial, in the monkey study of Marino and Mazer (2018) the attention pointer has only been cued and trained in initial instruction trials. During the task, monkeys had to establish this pointer by themselves. Second, endogenous attention from memory is a resource demanding task (Schreij et al. 2008; Grubb et al. 2015; Demeter and Woldorff 2016). Third, probes used for mapping the state of attention were low contrast but nevertheless quite salient and may distract attention. Forth, saccade planning may also interfere with the task of keeping an endogenous attention pointer in working memory as the maintenance of an attention pointer involves overlapping brain areas to those involved in saccade preparation. For example, stable remapping has been observed when a strong attentional cue is presented sufficiently early prior to saccade onset (Szinte et al. 2018). Finally, different experimental instructions might prompt subjects to optimize their behavior in different ways. Whereas the studies with humans instructed participants to respond to all stimuli irrespective of their location, the monkeys were not rewarded when they responded to a target stimulus appearing at a location different from the instructed one. Under these conditions, lingering attention may be detrimental to overall performance, which could lead to a strategy aimed at reducing post‐saccadic attentional spread by withdrawing the attention pointer during saccade planning. This conclusion is supported by our finding that the experimental data were best fit when the attention pointer was removed in the model between saccade‐target onset and saccade onset. In summary, our model suggests spatial remapping of attention pointers in LIP as a likely cause of attention pointer updating in V4 during eye movements. Our results emphasize the interplay of parietal and ventral brain areas for the subjective experience of a visually stable world around eye movements.

## Author Contributions

**Julia Bergelt:** data curation (lead), formal analysis (lead), methodology (equal), software (lead), validation (lead), visualization (lead), writing (lead), review and editing (supporting). **Fred H. Hamker:** conceptualization (lead), funding acquisition (lead), project administration (lead), resources (lead), supervision (lead), writing (supporting), review and editing (lead).

## Funding

This work was supported by the Federal Ministry of Education and Research (01GQ1409) and the Deutsche Forschungsgemeinschaft (HA 2630/15‐1).

## Conflicts of Interest

The authors declare no conflicts of interest.

## Supporting information


**Figure S1:** Structure of the neuro‐computational model consisting of V1, V4 with Layer 4 and Layer 2/3, FEF with visual, visuomovement, and movement cells (FEFv, FEFvm, and FEFm, respectively), LIP split according to corresponding input signal (CD signal and PC signal, respectively), and an intermediate map Xh. Inputs: visual presentation, proprioceptive eye position, saccade planning, and top‐down attention pointer. The different maps (and inputs) are operating in different reference frames: V1, V4, and FEF as well as the visual input and saccade planning are retinotopic organized (visualized as parallelograms); PC, CD, and the top‐down attention signal are in head‐centered coordinates (circles), and both LIP maps operate in a combined retinotopic‐head‐centered reference frame (depicted as octagon).
**Figure S2:** Temporal dynamics of the four input signals (signal^vis^, signal^att^, signal^PC^, signal^CD^). As the saccade is only horizontal, the vertical component can be neglected and is therefore fixed (horizontal center line of the visual array for signal^PC^ and signal^CD^, above the horizontal center line for signal^att^, and above and below horizontal center line for signal^vis^ top and bottom, respectively). The time (in milliseconds) is aligned to saccade onset. The bottom row depicts the spatial layout including fixation point (FP), saccade target (ST), attention position (AP), and presented stimuli (gray bars).
**Table S1:** Measurement of distance depending on dimension of the pre‐ and postsynaptic neurons and the way how the maps are connected. The last column indicates, in which equation the connection pattern is used.
**Table S2:** Parameters for input signals.
**Table S3:** Dimensions of different maps.
**Table S4:** Parameters for ODEs and connections. Bold parameters were varied in the robustness analysis.

**Video S1:** Simulated experimental setup as well as firing rates of all important populations of the neuro‐computational over time. The layout of the maps follows Figure S1.

## Data Availability

All data has been produced on the basis of our neurocomputational model. We made all files required to reproduce the data publicly available at https://github.com/hamkerlab/Bergelt2025_AttentionalUpdatingInV4. The model is implemented in Python 3.10 and is simulated with the neural simulator ANNarchy 4.8.2 (Vitay et al. 2015).

## Endnotes

^1^
As the saccades were exclusively directed towards the right, we can plot the activity with respect only to the horizontal position of space.

^2^
The V4 activity patterns resulting from simulations with only one (fixed) attention position are quantitatively the same as with four attention positions. The results are only not as smooth as with the full setup because not all possible combinations of bar position and onset are simulated.
