# Supplementary material for: Perisaccadic Attentional Updating in Area V4: A Neurocomputational Approach
Source: Eur J Neurosci. 2025 Dec 17;62(12):e70354. doi: 10.1111/ejn.70354 (PMC12712554; doi:10.1111/ejn.70354)
Supplement: Supplementary file 1 — Figure S1: Structure of the neuro‐computational model consisting of V1, V4 with Layer 4 and Layer 2/3, FEF with visual, visuomovement, and movement cells (FEFv, FEFvm, and FEFm, respectively), LIP split according to corresponding input signal (CD signal and PC signal, respectively), and an intermediate map Xh. Inputs: visual presentation, proprioceptive eye position, saccade planning, and top‐down attention pointer. The different maps (and inputs) are operating in different reference frames: V1, V4, and FEF as well as the visual input and saccade planning are retinotopic organized (visualized as parallelograms); PC, CD, and the top‐down attention signal are in head‐centered coordinates (circles), and both LIP maps operate in a combined retinotopic‐head‐centered reference frame (depicted as octagon). Figure S2: Temporal dynamics of the four input signals (signalvis, signalatt, signalPC, signalCD). As the saccade is only horizontal, the vertical component can be neglected and is therefore fixed (horizontal center line of the visual array for signalPC and signalCD, above the horizontal center line for signalatt, and above and below horizontal center line for signalvis top and bottom, respectively). The time (in milliseconds) is aligned to saccade onset. The bottom row depicts the spatial layout including fixation point (FP), saccade target (ST), attention position (AP), and presented stimuli (gray bars). Table S1: Measurement of distance depending on dimension of the pre‐ and postsynaptic neurons and the way how the maps are connected. The last column indicates, in which equation the connection pattern is used. Table S2: Parameters for input signals. Table S3: Dimensions of different maps. Table S4: Parameters for ODEs and connections. Bold parameters were varied in the robustness analysis. [file EJN-62-0-s001.pdf]

Supplementing Material

Neuro-computational model

The neuro-computational model is depicted in detail in Figure S1. It receives 4 different inputs and consists of 11 maps: Primary visual cortex *V1*, fourth visual cortex V4 split into *V4, Layer 4* and *V4, Layer 2/3*, frontal eye field FEF divided into visual (*FEFv*), visuomovement (*FEFvm*), and movement (*FEFm*) cells, two-eye related maps modeling either corollary discharge (*CD signal*) or proprioceptive eye position (*PC signal*), lateral intraparietal area LIP split into CD and PC driven cells (*LIP CD* and *LIP PC*, respectively), and an (artificial) intermediate map *Xh* for the interaction between the two LIP maps. The spatial layout of each map consists of a horizontal and a vertical dimension. Both LIP maps consist of two two-dimensional spatial dimensions resulting in four-dimensional gain-field maps each. The connections between the maps are presented by the arrows in Fig. S1 and in the mathematical description of the maps (see sections ODEs and Connections).

Inputs

Inputs are the *visual presentation* containing a continuous stream of bars, the *proprioceptive eye position* over time updating from fixation (FP) to saccade target (ST), the saccade target itself including temporal component of rise and decay comparable to *corollary discharge*, and a *top-down attention pointer* towards the attention position (AP).

The input image with its bars flashing at different timesteps for different durations is transferred into a *visual signal* defined through Gaussian receptive fields stimulated by the presented bars. The visual signal over time for a neuron n is given by:

$$signal^{\text{vis}}(t,n) = K^{\text{vis}} \sum_{sp \in stimuli} TC^{\text{vis}}(t,sp) \exp \frac{-\|sp - c^{\text{vis}}(n)\|^2}{2(\sigma^{\text{vis}})^2} \tag{1}$$

with K^{vis} and σ^{vis} the strength and width of the signal and $\|sp - c^{\text{vis}}(n)\|^2$ the distance between stimulus position sp and receptive field center $c^{\text{vis}}(n)$ of neuron n summed over all presented stimuli. $TC^{\text{vis}}(t,sp)$

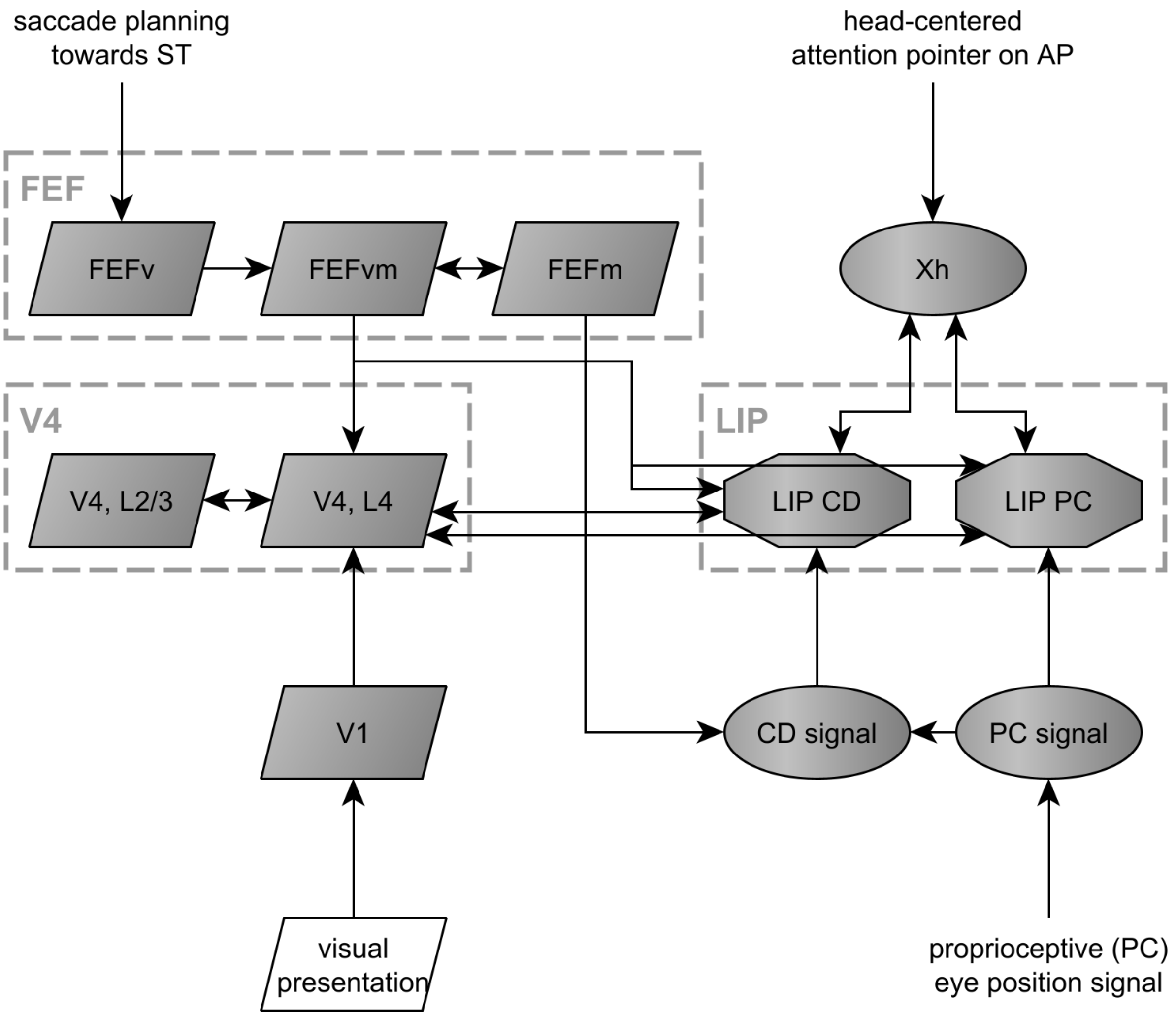

Figure S1: Structure of the neuro-computational model consisting of V1, V4 with Layer 4 and Layer 2/3, FEF with visual, visuomovement and movement cells (FEFv, FEFvm and FEFm, respectively), LIP split according to corresponding input signal (CD signal and PC signal, respectively) and an intermediate map Xh. Inputs: visual presentation, proprioceptive eye position, saccade planning, and top-down attention pointer.The different maps (and inputs) are operating in different reference frames: V1, V4, and FEF as well as the visual input and saccade planning are retinotopic organized (visualized as parallelograms), PC, CD and the top-down attention signal are in head-centered coordinates (circles), and both LIP maps operate in a combined retinotopic-head-centered reference frame (depicted as octagon).

represents the time course of a stimulus sp, i.e. the specific time when it is presented, and is defined as:

$$TC^{\text{vis}}(t, sp) = \begin{cases} 1, & \text{if stimulus } sp \text{ is presented at time } t \\ 0, & \text{else} \end{cases}$$

The *top-down attention signal* is modeled as a Gaussian function:

$$signal^{\text{att}}(t,n) = K^{\text{att}} \exp \frac{-\|ap - c^{\text{att}}(n)\|^2}{2(\sigma^{\text{att}})^2} \tag{2}$$

with K^{att} and σ^{att} the strength and width of the signal and $\|ap - c^{\text{att}}(n)\|^2$ the distance between attended position ap and receptive field center $c^{\text{att}}(n)$ of neuron n.

The *(proprioceptive) eye position* is modeled by a Gaussian function centered first at the fixation point FP and afterwards at the saccade target ST including a delayed updating:

$$signal^{\text{PC}}(t,n) = K^{\text{PC}} \sum_{ep \in \{FP,ST\}} TC^{\text{PC}}(t,ep) \exp \frac{-\|ep - c^{\text{PC}}(n)\|^2}{2(\sigma^{\text{PC}})^2} \tag{3}$$

with K^{PC} and σ^{PC} the strength and width of the signal and $\|ep - c^{\text{PC}}(n)\|^2$ the distance between eye position position ep and receptive field center $c^{\text{PC}}(n)$ of neuron n summed over pre- and postsaccadic eye position (i. e. fixation point and saccade target). After a saccade, the activity at FP decays while the activity at ST rises. As the PC signal is sluggish, the update from fixation point to saccade target is delayed. Furthermore, the presaccadic activity at FP will not be switched off immediately, but fades out with a Gaussian decay rate:

$$TC^{\text{PC}}(t,ep) = \begin{cases} \exp \frac{-\left(t - t^{PC_{update}}\right)^2}{2\left(\sigma^{PC_{decay}}\right)^2}, & \text{if } t \geq t^{PC_{update}} \text{ and } ep = FP \\ 0, & \text{if } t < t^{PC_{update}} \text{ and } ep = ST \\ 1, & \text{else} \end{cases}$$

The input to neuron n of *FEFv* is a Gaussian-shaped population centered at the saccade target with a temporal component of rising and decay mimicking a corollary discharge. Note, that FEF is organized in an eye-centered reference frame, thus the center of the input's receptive field is not the (head-centered) saccade target, but the saccade vector. The input is defined as:

$$signal^{\text{CD}}(t,n) = TC^{\text{CD}}(t) K^{\text{CD}} \exp \frac{-\|st - c^{\text{CD}}(n)\|^2}{2(\sigma^{\text{CD}})^2} \tag{4}$$

with K^{CD} and σ^{CD} the strength and width of the signal and $\|st - c^{\text{CD}}(n)\|^2$ the distance between (eye-centered) saccade target st and receptive field center $c^{\text{CD}}(n)$ of neuron n. $TC^{\text{CD}}(t)$ defines the temporal

rise and decay of the signal dependent on the timestep of saccade onset:

$$TC^{\mathrm{CD}}(t) = \begin{cases} \exp \frac{-\left(t - t^{CD_{peak}}\right)^2}{2\left(\sigma^{CD_{rise}}\right)^2}, & \text{if } t \leq t^{CD_{peak}} \\ \exp \frac{-\left(t - t^{CD_{peak}}\right)^2}{2\left(\sigma^{CD_{decay}}\right)^2}, & \text{else} \end{cases}$$

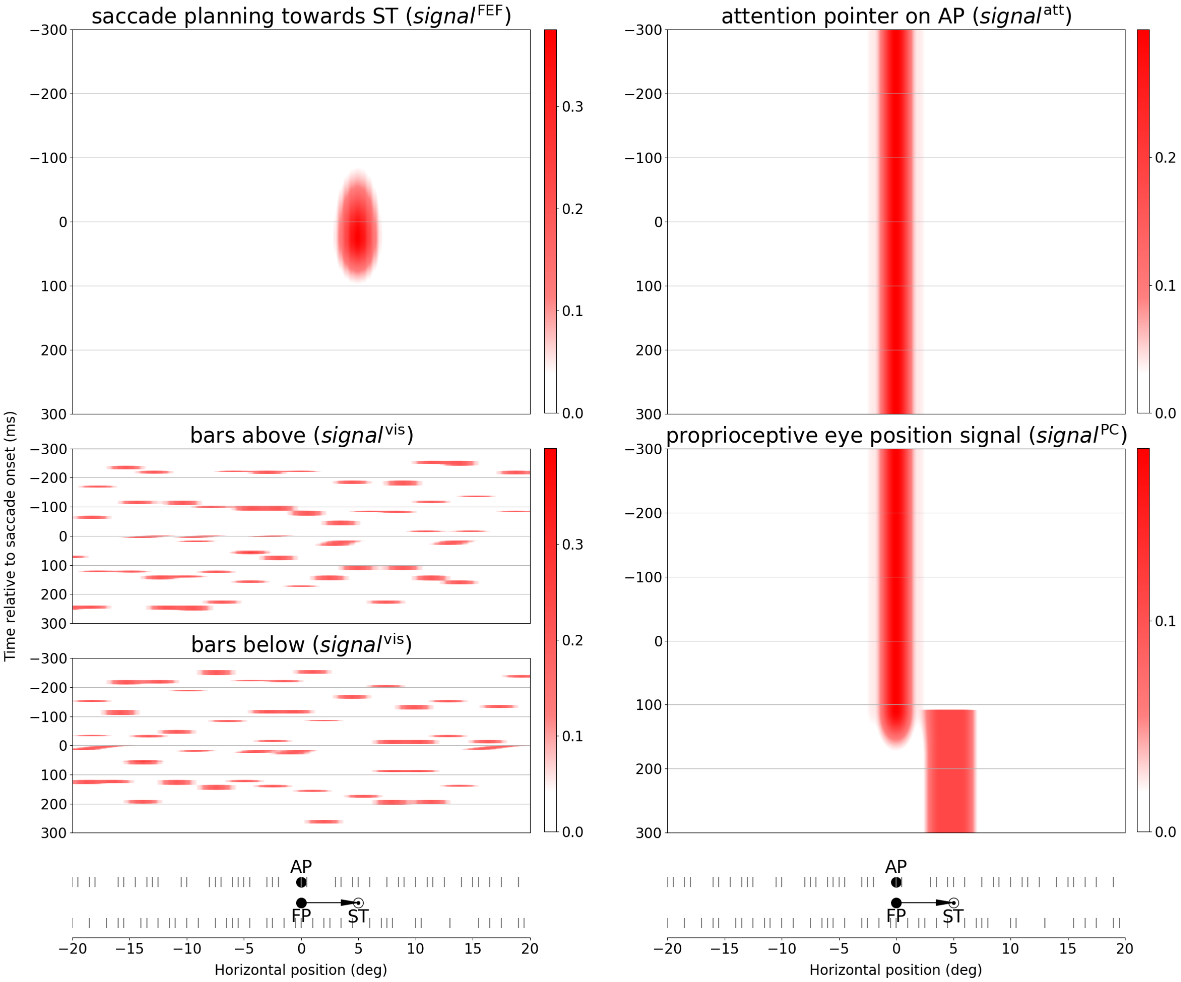

Figure S2: Temporal dynamics of the four input signals ($signal^{\mathrm{vis}}$, $signal^{\mathrm{att}}$, $signal^{\mathrm{PC}}$, $signal^{\mathrm{CD}}$). As the saccade is only horizontal, the vertical component can be neglected and is therefore fixed (horizontal center line of the visual array for $signal^{\mathrm{PC}}$ and $signal^{\mathrm{CD}}$, above the horizontal center line for $signal^{\mathrm{att}}$, and above and below horizontal center line for $signal^{\mathrm{vis}}$ top and bottom, respectively). The time (in milliseconds) is aligned to saccade onset. The bottom row depicts the spatial layout including fixation point (FP), saccade target (ST), attention position (AP) and presented stimuli (gray bars).

ODEs

The model consists of different maps, each with multiple rate-coded neurons. The firing rates of the neurons are computed by ordinary differential equations (ODE).

The ODEs of the three maps *V1*, *PC signal* and *Xh* processing the inputs *visual signal* derived from the *visual presentation*, *(proprioceptive) eye position* and *top-down attention* are defined as:

$$\tau \frac{d}{dt} r^{*}(t,n) = [signal^{*}(t,n)]^{+} - r(t,n) \tag{5}$$

where $signal^{*}$ is defined according to the actual input signal for the map ($signal^{\mathrm{vis}}$ for *V1*, $signal^{\mathrm{PC}}$ for *PC signal*, and $signal^{\mathrm{att}}$ for *Xh*; see above) and $[...]^{+}$ a positive function allowing only non-negative inputs. The firing rates of the neurons in *FEFv* do not follow an ODE, but are simply set to the input signal $signal^{\mathrm{FEF}}$.

The activity of *FEFv* is sent to the visuomovement cells *FEFvm*. This map is split into several planes realizing competition between different locations. *FEFvm* projects its activity to *FEFm* where the saccade target is encoded in a retinotopic reference frame. This information is fed back to *FEFvm* to enhance this position and suppress other locations. Additionally, there is a binary fixation cell *FEFfix* which can suppress saccade executions (e. g. to avoid a consecutive saccade following too early). The ODEs of these two FEF maps are defined as follows:

$$\begin{aligned}\tau \frac{d}{dt} r^{FEFvm}(t,n) = v^{FEFvm} &\left(v^{\mathrm{FEFv}} \left[E^{FEFv}(t,n)\right]^{+} + \left(1 - v^{\mathrm{FEFv}}\right) \left[E^{FEFv}(t,n) - S^{FEFv}(t,n)\right]_{0}^{1} \right) \\ &+ (1 - v^{FEFvm}) r^{FEFm}(t,n) - r^{FEFvm}(t,n) \qquad (6) \\ \tau \frac{d}{dt} r^{FEFm}(t,n) = E^{FEFvm}&(t,n) - S^{FEFvm}(t,n) - S^{Fix}(t,n) - r^{FEFm}(t,n) \qquad (7)\end{aligned}$$

with $[...]^{+}$ the positive function as above, $[...]_{0}^{1}$ a clipping function bounding the values between 0 and 1 and v^{FEFvm} a factor enabling different weightings between excitation and suppression for each plane

independently. The excitations (E^*) and suppressions (S^*) are defined as follows:

$$
\begin{aligned}
E^{FEFv}(t,n) &= v^{FEFv,exc} sum(FEFv, exc) \\
S^{FEFv}(t,n) &= v^{FEFv,sup} sum(FEFv, sup) \\
E^{FEFvm}(t,n) &= v^{FEFvm,exc} \overline{r}^{FEFvm}(t,n) \\
S^{FEFvm}(t,n) &= v^{FEFvm,sup} \max_{n'} \left(\overline{r}^{FEFvm}(t,n') \right) \\
S^{Fix}(t,n) &= v^{FEFfix} r^{FEFfix}(t)
\end{aligned}
$$

where $\overline{r}^{FEFvm}(t,n)$ is the mean of $r^{FEFvm}(t,n)$ over all planes and $sum(FEFv,*)$ a weighted sum between map *FEFv* and *FEFvm* (both defined es convolutions where the absolute values of the weights of excitation and suppression are identical, but the sign is flipped).

The visual input is transferred from *V1* to *V4, Layer 4*. Additionally, *V4, Layer 4* receives spatial amplification from *FEFvm* and both LIP maps as well as feature-based attention from *V4, Layer 2/3*:

$$
\tau \frac{d}{dt} r^{V4L4}(t,n) = v^{V4L4} \frac{E(t,n) \cdot A(t,n)}{\sigma^{V4L4} + E(t,n) \cdot A(t,n)} - r^{V4L4}(t,n) \tag{8}
$$

with

$$
E(t,n) = \left(v^{V1} \left[sum(V1) \right]_0^1 \right)^{p^{V1}}
$$

the excitatory input from *V1* and

$$
A(t,n) = 1.0 + \underbrace{v^{FEFvm} \overline{r}^{FEFvm}(t,n)}_{\text{sp. att. from } FEFvm} + \underbrace{v^{V4L23} \left(sum(V4L23) \right)^{p^{V4L23}}}_{\text{fb. att. from } V4,\ L2/3} + \underbrace{v^{LIP} \left(sum(LIPcd) + sum(LIPpc) \right)}_{\text{sp. att. from LIP}}
$$

the amplification where $sum(*)$ is a weighted sum between pre-map $*$ and *V4, Layer 4* and $[...]_0^1$ the clipping function as above. Due to the simplicity of the visual input (uniform bars), we do not need complex visual maps and therefore no complex connection between *V1* and *V4, Layer 4*. Thus, the weighted sum $sum(V1)$ is reduced to a simple one-to-one connection. The connection from *V4, Layer 2/3* is defined as convolution while the connections from LIP are defined with a Gaussian pattern.

The firing rates in the pooling map *V4, Layer 2/3* are driven by *V4, Layer 4*:

$$
\tau \frac{d}{dt} r^{V4L23}(t,n) = v^{V4L23} \frac{E(t,n)}{\sigma^{V4L23} + E(t,n)} - r^{V4L23}(t,n) \tag{9}
$$

with

$$E(t,n) = \left(v^{V4L4} sum(V4L4)\right)^{p^{V4L4}}$$

the excitatory input from *V4, Layer 4* defined through the weighted sum $sum(V4L4)$ with a convolution.

Both LIP maps, *LIP CD* and *LIP PC*, receive visual input from *V4, Layer 4*, endogenous spatial attention from *Xh* and transient attention from the saccade plan through *FEFvm* as well as an eye-related input, either corollary discharge from *CD signal* or (proprioceptive) eye position from *PC signal*. As the eye-related input must be in a head-centered reference frame, the corollary discharge cannot be used directly from *FEFm* and therefore has to be transformed into a head-centered coordinate system combining *FEFm* with the *PC signal*:

$$\tau \frac{d}{dt} r^{CD}(t,n) = sum(FEFm) sum(PC) - \underbrace{r^{CD}(t,n) \cdot v^{CD} \sum_{n'} r^{CD}(t,n')}_{\text{inhibition}} - r^{CD}(t,n) \tag{10}$$

with $sum(*)$ the weighted sums from pre-map $*$ to *CD signal* defined with Gaussian patterns.

The ODEs for the LIP maps are defined as:

$$\begin{aligned}
\tau \frac{d}{dt} r^{LIPcd}(t,n) = {} & \underbrace{(sum(V4L4) + sum(FEFvm)) \left(0.1 + \left[A^{LIPcd} - r^{LIPcd}(t,n)\right]^{+} sum(CD)\right)}_{\text{feedforward input from } V4, L4 \text{ and } FEFvm} \\
& + \underbrace{norm\left(sum(Xh)\right) sum(CD)}_{\text{feedback from } Xh} - \underbrace{\left(r^{LIPcd}(t,n) + D^{LIPcd}\right) v^{LIPcd} \sum_{n'} r^{LIPcd}(t,n')}_{\text{inhibition}} \\
& - r^{LIPcd}(t,n) && (11) \\
\tau \frac{d}{dt} r^{LIPpc}(t,n) = {} & \underbrace{(sum(V4L4) + sum(FEFvm)) \left[A^{LIPpc} - \max_{n'} \left(r^{LIPpc}(t,n')\right)\right]^{+} sum(PC)}_{\text{feedforward input from } V4, L4 \text{ and } FEFvm} \\
& + \underbrace{norm\left(sum(Xh)\right) sum(PC)}_{\text{feedback from } Xh} - \underbrace{\left(r^{LIPpc}(t,n) + D^{LIPpc}\right) v^{LIPpc} \sum_{n'} r^{LIPpc}(t,n')}_{\text{inhibition}} \\
& + \underbrace{sum(exc)}_{\text{excitation}} - r^{LIPpc}(t,n) && (12)
\end{aligned}$$

with $sum(exc)$ lateral excitation, the remaining $sum(*)$ the weighted sums from pre-map $*$ to the respec-

tive LIP map (all defined with Gaussian patterns) and $norm(...)$ a divisive normalization:

$$norm(x) = \frac{x}{\sigma + x}$$

The LIP maps interact with each other via the intermediate map *Xh*. Thus, in addition to the *top-down attention*, *Xh* receives input from *LIP CD* and *LIP PC* and the ODE for *Xh* mentioned above must be extended:

$$\tau \frac{d}{dt} r^{Xh}(t,n) = \underbrace{norm\left(input(t,n)\right) S(t,n)}_{\text{input}} + \underbrace{sum(exc)}_{\text{excitation}} \\ - \underbrace{\left(r^{Xh}(t,n) + D^{Xh}\right) v^{Xh} \sum_{n'} r^{Xh}(t,n')}_{\text{inhibition}} - r^{Xh}(t,n) \tag{13}$$

with $sum(exc)$ lateral excitation defined with a Gaussian pattern, $norm(...)$ the divisive normalization defined above and

$$\begin{aligned} S(t,n) &= 1 - d^{Xh,dep} s(t,n) \\ \tau^{Xh,dep} \frac{d}{dt} s(t,n) &= input(t,n) - s(t,n) \end{aligned}$$

modeling synaptic suppression. The input is extended to

$$input(t,n) = sum(LIPcd) + sum(LIPpc) + signal^{att}(t,n)$$

with $sum(LIP*)$ the weighted sums from each LIP map to *Xh* defined with Gaussian patterns.

Connections

Most of the ODEs defined above make use of weighted sums $sum(*)$ to determine the synaptic transmission between two maps. Each connection follows a Convolution or a Gaussian pattern.

Convolutions A convolution is a weighted sum over a receptive field (RF) defined in the *pre* map. It is defined as a 2D Gaussian G with RF size $RF^{pre \to post}_{size}$ and width $RF^{pre \to post}_{\sigma} = [\sigma_1, \sigma_2]$:

$$G(x, RF_{\sigma}^{pre\to post}) = \exp\left(-\left(\frac{x_1 - x_1'}{2\sigma_1^2} + \frac{x_2 - x_2'}{2\sigma_2^2}\right)\right)$$

for $x = [x_1, x_2] \in RF_{size}^{pre\to post}$ and $x' = [x_1', x_2']$ the center of the RF. Additionally, the Gaussian can be scaled individually:

$$v^{pre\to post,factor}\left(G(x, RF_{\sigma}^{pre\to post}) - v^{pre\to post,scale}\right)$$

Gaussian patterns The Gaussian patterns must be defined in detail depending on the actual dimensions of the connected maps. Two neurons n' of *pre* map and n of *post* map are connected with the weighted sum $sum(pre)$ in an ODE of a *post* map:

$$sum(pre) = sum(pre, t, n) = \sum_{n'} w_{n'\to n}^{pre\to post} r^{pre}(t, n')$$

The weights $w_{n'\to n}^{pre\to post}$ follow a Gaussian function dependent on the distance *dist* of each pair of neurons (n', n):

$$w_{n'\to n}^{pre\to post} = K^{pre\to post}\exp\left(-\frac{dist(n', n)}{\sigma^{pre\to post}}\right)$$

Similarly, the lateral excitation in (12) and (13) is defined as:

$$sum(exc) = sum(exc, map, t, n) = \sum_{n'} w_{n'\to n}^{map,exc} r^{map}(t, n')$$

with

$$w_{n'\to n}^{map,exc} = K^{map,exc}\exp\left(-\frac{dist(n', n)}{\sigma^{map,exc}}\right)$$

and *map* either *LIP PC* or *Xh*.

Dependent on the dimension of the pre- and postsynaptic neurons (i.e. 2d, 3d or 4d), the way how the maps are connected, and with it the measurement of the distance $dist(n', n)$, varies. The different definitions are listed in Table S1.

Parameters

Next to the parameters defining the spatial and temporal layout of the experimental setup, the neuro-computational model consists of 77 different parameters used in the ODEs or to define the connections

pre neuron $n' \to$ post neuron n $(i',j'),(i,j) \in 2d$, $(i',j',p'),(i,j,p) \in 3d$ $(i',j',k',l'),(i,j,k,l) \in 4d$	measurement of $dist(n',n)$	used in Equation
$(i',j') \to (i,j)$	$\|i'-i\|^2 + \|j'-j\|^2$	(13)
$(i',j') \to (i,j,k,l)$, horizontal		(10), (11), (12)
$(i',j',p') \to (i,j,p)$		(6), (8), (9)
$(i',j',p') \to (i,j,k,l)$, horizontal		(11), (12)
$(i',j',k',l') \to (i,j,p)$, horizontal		(8)
$(i',j') \to (i,j,k,l)$, vertical	$\|i'-k\|^2 + \|j'-l\|^2$	(10), (12)
$(i',j') \to (i,j,k,l)$, diagonal	$\|i'-i-k\|^2 + \|j'-j-l\|^2$	(11), (12)
$(i',j',k',l') \to (i,j)$, diagonal	$\|i'+k'-i\|^2 + \|j'+l'-j\|^2$	(13)
$(i',j',k',l') \to (i,j,k,l)$, vertical	$\|i'+k'-k\|^2 + \|j'+l'-l\|^2$	(11)
$(i',j',k',l') \to (i,j,k,l)$	$\|i'-i\|^2 + \|j'-j\|^2 + \|k'-k\|^2 + \|l'-l\|^2$	(12)

Table S1: Measurement of distance depending on dimension of the pre- and postsynaptic neurons and the way how the maps are connected. The last column indicates, in which equation the connection pattern is used.

between the maps. All parameters used in the simulations are listed in the following tables, split for parameters regarding input signal (Table S2), dimensions of maps (Table S3) and model structure (Table S4).

The parameters used to define the input signals *visual signal*, *top-down attention signal*, *(proprioceptive) eye position*, and *FEFv* (see Equations (1) to (4)) are:

parameter name	value	used in Equation
K^{CD}	0.375	(4)
σ^{CD}	1.0	(4)
$\sigma^{CD_{rise}}$	60	(4)
$\sigma^{CD_{decay}}$	40	(4)
$t^{CD_{peak}}$	29	(4)
K^{PC}	0.3	(3)
σ^{PC}	1.0	(3)
$t^{PC_{update}}$	60	(3)
$\sigma^{PC_{decay}}$	35	(3)

K^{vis}	0.2	(1)
σ^{vis}	1.0	(1)
K^{att}	0.3	(2)
σ^{att}	1.0	(2)

Table S2: Parameters for input signals.

The visual field is defined in its horizontal and vertical dimension with $[40° \times 30°]$. Derived from this, the number of neurons in the different maps are defined. The used values including an interpretation of the different dimensions are listed in the following table:

map	dimension	interpretation
V1	$(81, 61, 1, 1)$	(horizontal, vertical, channels, planes)
V4, Layer 4	$(81, 61, 1)$	(horizontal, vertical, planes)
V4, Layer 2/3	$(41, 31, 1)$	(horizontal, vertical, planes)
FEFv, *FEFm*	$(81, 61)$	(horizontal, vertical)
FEFvm	$(81, 61, 6)$	(horizontal, vertical, planes)
PC signal, *Xh*	$(21, 16)$	(horizontal, vertical)
CD signal, *LIP PC*, *LIP CD*	$(21, 16, 21, 16)$	(horizontal, vertical, horizontal, vertical)

Table S3: Dimensions of different maps.

Finally, the parameters used in the ODEs (Equations (5) to (13), first part of table) and the parameters used to define the connection patterns (second part of table) are listed below. The bold parameters were varied to test the robustness of the model.

parameter name	value	used in Equation
$\boldsymbol{\sigma}^{\mathbf{V4L4}}$	**0.4**	**(8)**
$\mathbf{v}^{\mathbf{V4L4}}$	**1.066**	**(8)**
$\mathbf{v}^{\mathbf{V1}}$	**1.0**	**(8)**
$\mathbf{p}^{\mathbf{V1}}$	**1**	**(8)**
$\mathbf{v}^{\mathbf{V4L23}}$	**0.5**	**(8)**

$\mathbf{p^{V4L23}}$	**1**	**(8)**
$\mathbf{v^{FEFvm}}$	**4.0**	**(8)**
$\mathbf{v^{LIP}}$	**3.0**	**(8)**
$\boldsymbol{\sigma}^{\mathbf{V4L23}}$	**1.0**	**(9)**
$\mathbf{v^{V4L23}}$	**1.625**	**(9)**
$\mathbf{v^{V4L4}}$	**1.0**	**(9)**
$\mathbf{p^{V4L4}}$	**0.25**	**(9)**
v^{FEFv}	0.2	(6)
$v^{FEFv,exc}$	0.6	(6)
$v^{FEFv,sup}$	0.6	(6)
$v^{FEFvm,exc}$	1.3	(7)
$v^{FEFvm,sup}$	0.3	(7)
v^{FEFfix}	0.3	(7)
$\mathbf{A^{LIPcd}}$	**0.5**	**(11)**
$\mathbf{A^{LIPpc}}$	**1.0**	**(12)**
$\mathbf{D^{LIPcd}}$	**0.1**	**(11)**
$\mathbf{D^{LIPpc}}$	**0.1**	**(12)**
$\mathbf{D^{Xh}}$	**0.6**	**(13)**
$\boldsymbol{\sigma}^{\mathbf{LIPcd}}$	**0.5**	**(11)**
$\boldsymbol{\sigma}^{\mathbf{LIPpc}}$	**1.0**	**(12)**
$\boldsymbol{\sigma}^{\mathbf{Xh}}$	**0.5**	**(13)**
$\mathbf{d^{Xh,dep}}$	**2.2**	**(13)**
$\tau^{Xh,dep}$	10000	(13)
τ	10	(5) to (13)
$\mathbf{v^{CD}}$	**0.05**	**(10)**
$\mathbf{v^{LIPcd}}$	**0.05**	**(11)**
$\mathbf{v^{LIPpc}}$	**0.2**	**(12)**
$\mathbf{v^{Xh}}$	**1.0**	**(13)**
v^{FEFvm}	[1. 0.8 0.6 0.4 0.2 0.]	(6)
$\mathbf{delay^{V1 \rightarrow V4L4}}$	**5**	**(8), sum(V1)**

$\mathbf{p^{V4L4 \to V4L23, ws}}$	**4**	**(9), sum(V4L4)**
$v^{FEFv \to FEFvm, scale}$	0.35	(6), $sum(FEFv)$
$v^{FEFv \to FEFvm, factor}$	0.93	(6), $sum(FEFv)$
$RF_{\sigma}^{V4L23 \to V4L4}$	[0.8436214 0.84699454]	(8), $sum(V4L23)$
$RF_{size}^{V4L23 \to V4L4}$	[3, 3]	(8), $sum(V4L23)$
$\mathbf{delay^{V4L23 \to V4L4}}$	**2**	**(8), sum(V4L23)**
$RF_{size}^{V4L4 \to V4L23}$	[5, 5]	(9), $sum(V4L4)$
$RF_{\sigma}^{V4L4 \to V4L23}$	[1.6666666666666667, 1.6666666666666667]	(9), $sum(V4L4)$
$RF_{size}^{FEFv \to FEFvm}$	[41, 31]	(6), $sum(FEFv)$
$RF_{\sigma}^{FEFv \to FEFvm}$	[4, 3]	(6), $sum(FEFv)$
$\mathbf{K^{FEFm \to CD}}$	**1.0**	**(10), sum(FEFm)**
$\mathbf{K^{PC \to CD}}$	**10.0**	**(10), sum(PC)**
$\mathbf{K^{FEFvm \to LIPcd}}$	**0.015**	**(11), sum(FEFvm)**
$\mathbf{K^{FEFvm \to LIPpc}}$	**0.1**	**(12), sum(FEFvm)**
$\mathbf{K^{V4L4 \to LIPcd}}$	**0.5**	**(11), sum(V4L4)**
$\mathbf{K^{V4L4 \to LIPpc}}$	**2.0**	**(12), sum(V4L4)**
$\mathbf{K^{CD \to LIPcd}}$	**4.0**	**(11), sum(CD)**
$\mathbf{K^{PC \to LIPpc}}$	**1.0**	**(12), sum(PC)**
$\mathbf{K^{Xh \to LIPcd}}$	**7.0**	**(11), sum(Xh)**
$\mathbf{K^{Xh \to LIPpc}}$	**2.0**	**(12), sum(Xh)**
$\mathbf{K^{LIPcd \to Xh}}$	**0.03**	**(13), sum(LIPcd)**
$\mathbf{K^{LIPpc \to Xh}}$	**0.05**	**(13), sum(LIPpc)**
$\mathbf{K^{LIPcd \to V4L4}}$	**0.5**	**(8), sum(LIPcd)**
$\mathbf{K^{LIPpc \to V4L4}}$	**0.2**	**(8), sum(LIPpc)**
$\mathbf{K^{LIPpc, exc}}$	**0.6**	**(12), sum(exc)**
$\mathbf{K^{Xh, exc}}$	**1.0**	**(13), sum(exc)**
$\boldsymbol{\sigma}^{\mathbf{FEFm \to CD}}$	**1.0**	**(10), sum(FEFm)**
$\boldsymbol{\sigma}^{\mathbf{PC \to CD}}$	**0.5**	**(10), sum(PC)**
$\boldsymbol{\sigma}^{\mathbf{FEFvm \to LIPcd}}$	**1.0**	**(11), sum(FEFvm)**
$\boldsymbol{\sigma}^{\mathbf{FEFvm \to LIPpc}}$	**1.0**	**(12), sum(FEFvm)**

$\boldsymbol{\sigma}^{\mathbf{V4L4 \to LIPcd}}$	**0.5**	**(11), sum(V4L4)**
$\boldsymbol{\sigma}^{\mathbf{V4L4 \to LIPpc}}$	**0.5**	**(12), sum(V4L4)**
$\boldsymbol{\sigma}^{\mathbf{CD \to LIPcd}}$	**0.25**	**(11), sum(CD)**
$\boldsymbol{\sigma}^{\mathbf{PC \to LIPpc}}$	**1.0**	**(12), sum(PC)**
$\boldsymbol{\sigma}^{\mathbf{Xh \to LIPcd}}$	**1.0**	**(11), sum(Xh)**
$\boldsymbol{\sigma}^{\mathbf{Xh \to LIPpc}}$	**1.0**	**(12), sum(Xh)**
$\boldsymbol{\sigma}^{\mathbf{LIPcd \to Xh}}$	**1.0**	**(13), sum(LIPcd)**
$\boldsymbol{\sigma}^{\mathbf{LIPpc \to Xh}}$	**1.0**	**(13), sum(LIPpc)**
$\boldsymbol{\sigma}^{\mathbf{LIPcd \to V4L4}}$	**0.5**	**(8), sum(LIPcd)**
$\boldsymbol{\sigma}^{\mathbf{LIPpc \to V4L4}}$	**0.5**	**(8), sum(LIPpc)**
$\boldsymbol{\sigma}^{\mathbf{LIPpc,exc}}$	**0.25**	**(12), sum(exc)**
$\boldsymbol{\sigma}^{\mathbf{Xh,exc}}$	**0.25**	**(13), sum(exc)**

Table S4: Parameters for ODEs and Connections. Bold parameters were varied in the robustness analysis.
